# Inhibition of poly(ADP-Ribosyl)ation reduced vascular smooth muscle cells loss and improves aortic disease in a mouse model of human accelerated aging syndrome

**DOI:** 10.1038/s41419-024-07078-7

**Published:** 2024-10-02

**Authors:** Déborah Cardoso, Solenn Guilbert, Philippe Guigue, Aurélie Carabalona, Karim Harhouri, Cécile Peccate, Johana Tournois, Zoheir Guesmia, Lino Ferreira, Catherine Bartoli, Nicolas Levy, Laurence Colleaux, Xavier Nissan, Antoine Muchir

**Affiliations:** 1 Sorbonne Université, UPMC Paris 06, INSERM UMRS974, Center of Research in Myology, Institut de Myologie, Paris, France; 2 Université Paris-Saclay, Université d’Evry, Inserm, IStem UMR861, Corbeil-Essonnes, France; 3 IStem, CECS, Corbeil-Essonnes, France; 4 Aix Marseille Université, INSERM, MMG, U1251 Faculté de Médecine Timone, Marseille, France; 5 Center for Neuroscience and Cell Biology, University of Coimbra, Coimbra, Portugal; 6 Faculty of Medicine, University of Coimbra, Coimbra, Portugal

**Keywords:** Aortic diseases, Target identification, Mechanisms of disease

## Abstract

Hutchinson-Gilford progeria syndrome (HGPS) is an extremely rare genetic disorder associated with features of accelerated aging. HGPS is an autosomal dominant disease caused by a de novo mutation of *LMNA* gene, encoding A-type lamins, resulting in the truncated form of pre-lamin A called progerin. While asymptomatic at birth, patients develop symptoms within the first year of life when they begin to display accelerated aging and suffer from growth retardation, and severe cardiovascular complications including loss of vascular smooth muscle cells (VSMCs). Recent works reported the loss of VSMCs as a major factor triggering atherosclerosis in HGPS. Here, we investigated the mechanisms by which progerin expression leads to massive VSMCs loss. Using aorta tissue and primary cultures of murine VSMCs from a mouse model of HGPS, we showed increased VSMCs death associated with increased poly(ADP-Ribosyl)ation. Poly(ADP-Ribosyl)ation is recognized as a post-translational protein modification that coordinates the repair at DNA damage sites. Poly-ADP-ribose polymerase (PARP) catalyzes protein poly(ADP-Ribosyl)ation by utilizing nicotinamide adenine dinucleotide (NAD^+^). Our results provided the first demonstration linking progerin accumulation, augmented poly(ADP-Ribosyl)ation and decreased nicotinamide adenine dinucleotide (NAD^+^) level in VSMCs. Using high-throughput screening on VSMCs differentiated from iPSCs from HGPS patients, we identified a new compound, trifluridine able to increase NAD^+^ levels through decrease of PARP-1 activity. Lastly, we demonstrate that trifluridine treatment in vivo was able to alleviate aortic VSMCs loss and clinical sign of progeria, suggesting a novel therapeutic approach of cardiovascular disease in progeria.

## Introduction

Hutchinson–Gilford progeria syndrome (HGPS) is a rare, to date incurable, genetic disorder characterized by early and accelerated aging. In its classical form, HGPS is a sporadic autosomal dominant disease caused by a point mutation in the *LMNA* gene *(*c.1824 C > T; p.G608G) [1, 2]. *LMNA* encodes for nuclear lamin A/C proteins generated by alternative splicing [3]. Lamin A is synthetized as a pre-lamin A, which is post-translationally modified by farnesylation at the C-terminal CaaX motif and subsequently cleaved by the metalloproteinase ZMPSTE24 to remove the farnesyl groups and produce mature lamin A [4]. The *LMNA* c.1824 C > T mutation activates a cryptic splice donor site, resulting in a mutant form of pre-lamin A with a 50-amino acid internal deletion (pre-lamin AΔ50; 606–656) encompassing the ZMPSTE24 cleavage site. This mutation leads to the synthesis of a shortened, incompletely processed, and constitutively farnesylated pre-lamin A, called progerin. The C-terminal CaaX motif of pre-lamin A undergoes methylation due to the activity of isoprenylcysteine carboxyl methyltransferase, therefore progerin remains also permanently carboxymethylated [5, 6]. Progerin accumulates in cell nuclei exerting multiple toxic effects on nuclear homeostasis, which cause premature senescence [7].

Although HGPS children have no symptoms at birth, they develop various clinical signs of ageing typically beginning in their first year or second years life. Patients suffer from growth retardation, bone and tendon abnormalities, lipodystrophy, alopecia, and severe cardiovascular complications including arterial fibrosis, loss of vascular smooth muscle cells (VSMCs), and severe arterial damage. Changes in both vascular architecture and pressure (systolic and diastolic) result in myocardial infarction, which often leads to premature death of HGPS patients at a mean age of 14.6 years [8]. Given that HGPS patients die prematurely from cardiovascular disease, a deeper understanding of the mechanisms through which progerin expression leads to premature cardiovascular pathology is essential to provide therapies for HGPS patients. Progressive loss of aortic VSMCs is characteristic of HGPS patients [9–11] and mouse models of progeria [12, 13]. Hence, alteration of VSMCs plays a key role in the development of cardiovascular disorders in mouse models of progeria, but the underlying mechanisms remain poorly understood.

VSMCs convert chemical energy from adenosine triphosphate (ATP) into mechanical activity, which is necessary for contractions. ATP production is dependent on nicotinamide adenine dinucleotide (NAD), a fundamental molecule that catalyzes electron transfer in metabolic reduction-oxidation reactions and exists in two forms, oxidized (NAD^+^) and reduced (NADH). In addition, NAD is also utilized as a co-substrate in a number of reactions including protein ADP-ribosylation by poly-ADP ribose polymerases (PARPs), biosynthesis of cyclic ADP-ribose by poly-adenosine diphospho-ribose (ADPR) synthases and deacetylation of lysines on histones by sirtuines (SIRTs). Accumulating evidence demonstrates an age-dependent decline of NAD^+^ levels and associates its depletion to several hallmarks of aging and age-related diseases [14], hence NAD emerges as potential new therapeutic target. PARPs are the major cellular NAD^+^ consumers by the catalysis of poly(ADP-Ribosyl)ation, a post-translational modification known to plays an important role in many processes related to aging and longevity such as chromatin organization, inflammation, maintenance of genomic integrity, transcription, and replication [15]. PARPs allows the transfer of multiple ADP-ribose groups from NAD^+^ to acceptor proteins, generating long poly(ADP-ribose) chains (PAR). NAD^+^ is therefore an essential co-substrate that can be synthetized from various precursors by three independent intracellular pathways, a de novo pathways, the Preiss-Handler pathway [16], and the salvage pathway [17]. Maintaining balance between NAD^+^ degradation and biosynthesis is therefore critical for cellular homeostasis.

In this study, we hypothesized that progerin expression could modify NAD^+^ homeostasis leading to VSMCs loss. We demonstrated reduced amount of NAD^+^ and increased levels of global poly(ADP-Ribosyl)ation in *Lmna*^*G609G/G609G*^ mice, a mouse model of HGPS. Using high-throughput screening on VSMCs differentiated from induced pluripotent stem cells (iPSCs) from HGPS patients, we identified trifluridine as a new compound able to restore NAD^+^ levels through decrease of PARP-1 activity. Treating *Lmna*^*G609G/G609G*^ mice with trifluridine led to a decreased loss of VSMCs and improvement of HGPS phenotype. These results further confirm the correlation between poly(ADP-Ribosyl)ation defect and progeroid phenotype and paves the way for new therapies targeting this pathway.

## Results

### Progerin accumulation correlates with activation of cell death programs in vascular smooth muscle cells

**Fig. 1:**
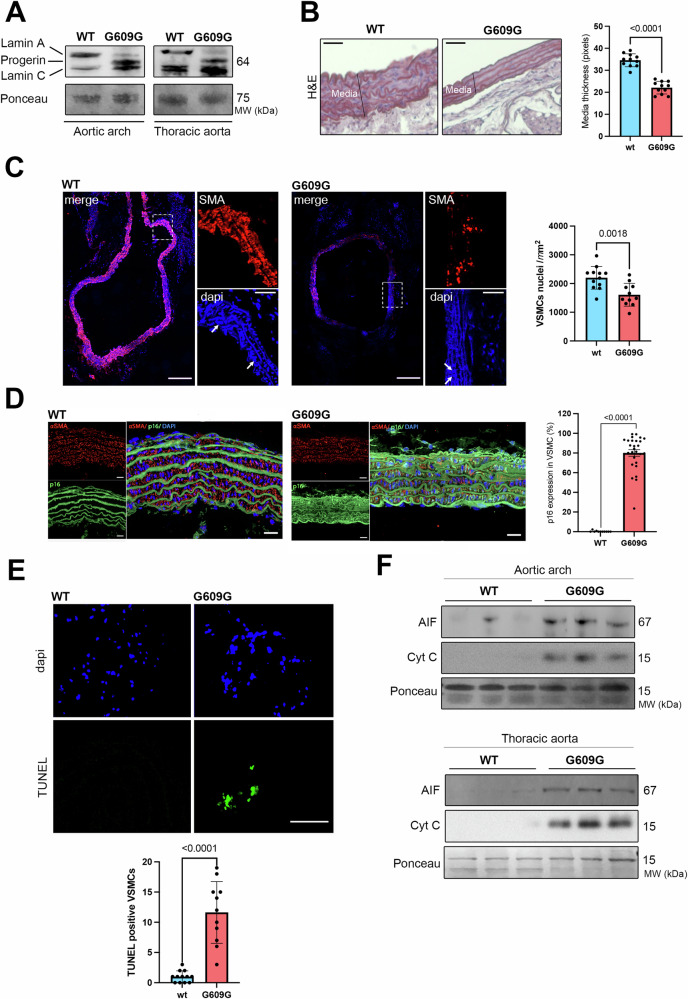
Increased aortic cell death in *Lmna*^*G609G/G609G*^ mice. **A** Representative immunoblots showing lamin A, progerin, and lamin C protein levels in aortic arch and thoracic aorta from *Lmna*^*G609G/G609G*^ (G609G) and WT mice. Ponceau was used for normalization. **B** Representative aorta cross sections from *Lmna*^*G609G/G609G*^ and WT mice stained with hematoxylin & eosin (H&E). Scale bar, 20 µm. Graph shows media thickness quantification in *Lmna*^*G609G/G609G*^ (*n* = 12) and WT (*n* = 11) mice. Bars indicate mean ± standard error of mean and numbers above columns indicate *p* values. Differences were analyzed by unpaired *t* test. **C** Immunostaining of aorta cross sections from *Lmna*^*G609G/G609G*^ and WT mice showing smooth muscle actin (SMA) (red) and nuclei stained with Hoechst (blue). The white arrows point to VSMCs nuclei. Left scale bars, 200 μm, and right scale bars, 40 µm. Graph shows VSMCs nuclei quantification in *Lmna*^*G609G/G609G*^ (*n* = 12) and WT (*n* = 11) mice. Bars indicate mean ± standard error of mean and numbers above columns indicate *p* values. Differences were analyzed by unpaired *t* test. **D** Immunostaining of aorta cross sections from *Lmna*^*G609G/G609G*^ (G609G) and WT mice showing α-SMA (red) and p16 (green) showing senescent cells. Nuclei stained with DAPI (blue). Scale bar, 20 µm. Graph shows the percentage of p16 senescent VSMCs in aortic arch quantification in *Lmna*^*G609G/G609G*^ (*n* = 6) and WT (*n* = 3) mice. Bars indicate mean ± standard error of mean and number above columns indicate *p* values. Differences were analyzed by unpaired *t* test. **E** TUNEL staining of aorta cross sections from *Lmna*^*G609G/G609G*^ and WT mice showing apoptotic cells (green). Scale bar, 40 µm. Graph shows TUNEL positive cells quantification in media from *Lmna*^*G609G/G609G*^ (*n* = 12) and WT (*n* = 11) mice. Bars indicate mean ± standard error of mean and numbers above columns indicate *p* values. Differences were analyzed by unpaired *t* test. **F** Immunoblots showing AIF and Cyt C protein levels in aortic arch and thoracic aorta from *Lmna*^*G609G/G609G*^ and WT mice. Ponceau was used for normalization.

We observed progerin expression in the aortic arch and thoracic aorta from 5 months old *Lmna*^*G609G/G609G*^ mice, which is a relevant animal model for studying HGPS [12] (Fig. 1A). *Lmna*^*G6096G/G6096G*^ mice showed decreased thickness of the media, the layer of the aortic wall containing VSMCs, as confirmed by hematoxylin/eosin staining, compared with control mice (Fig. 1B). A significant decreased number of smooth muscle actin-positive cells was also observed in *Lmna*^*G609G/G609G*^ mice, confirming a reduced number of VSMCs as shown previously [12, 13] (Fig. 1C). This was associated with a decreased senescence of VSMCs in the media of *Lmna*^*G609G/G609G*^ mice compared with control, as assessed with p16 positive cells (Fig. 1D). Given that oxidative stress is highly associated with cellular senescence, we next assessed the level of Nox2 in the media of *Lmna*^*G609G/G609G*^ mice. We showed a significant increased Nox2 expression in aorta from *Lmna*^*G609G/G609G*^ mice compared with control (Fig. S1). We next investigated if this increased oxidative stress in the aorta translated into inefficient DNA repair mechanism. We showed an increased expression of gamma-H2AX, an acute marker of DNA breaks, in aorta from *Lmna*^*G609G/G609G*^ mice compared with control (Fig. S2). Altogether, these results showed loss of VSMCs in the aorta from *Lmna*^*G609G/G609G*^ mice. To further study the underlying molecular mechanisms, we sought to investigate if apoptosis could be the trigger for the loss of VSMCs. A significant increased number of TUNEL-positive cells was observed in aorta from *Lmna*^*G609G/G609G*^ mice compared with control (Fig. 1E). Apoptosis can be mediated by a caspase-dependent pathway, which involves the mitochondrial release of cytochrome C (Cyt C) that initiates a cascade of caspase activation. In addition, there is a caspase-independent pathway, which is mediated by the release of apoptosis-inducing factor (AIF). Immunoblotting of proteins from both aortic arch and thoracic aorta indicated that Cyt C and AIF were significantly increased in *Lmna*^*G609G/G609G*^ mice compared with control (Fig. 1F). These results suggest that loss of VSMCs in HGPS is correlated with activation of cell death programs.

**Fig. 2:**
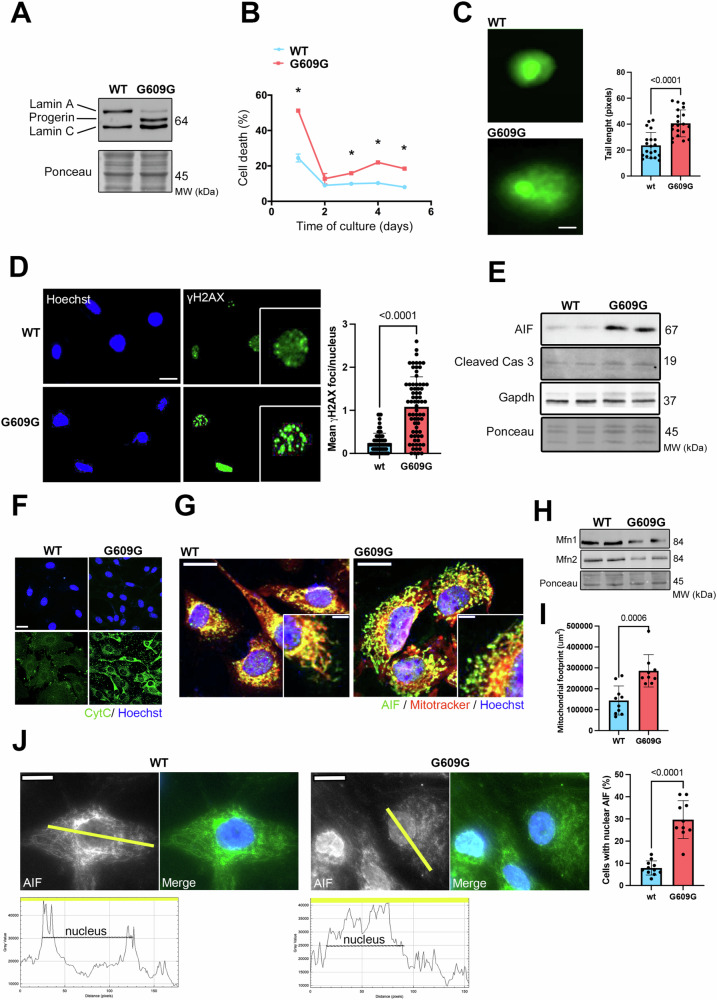
Increased apoptosis of cultured VSMCs from *Lmna*^*G609G/G609G*^*mice.* **A** Representative immunoblots showing lamin A, progerin, and lamin C protein levels in isolated VSMCs from *Lmna*^*G609G/G609G*^ (G609G) and WT mice. Ponceau was used for normalization. **B** Percentage of cell death curves of VSMCs from *Lmna*^*G609G/G609G*^ (G609G) (*n* = 3) and WT (*n* = 3) mice. Differences were analyzed by Kruskal–Wallis test, *p < 0.02. **C** Images of comets assay for VSMCs from *Lmna*^*G609G/G609G*^ (G609G) and WT mice. Scale bars, 10 µm. The graph shows tail length quantification for VSMCs from *Lmna*^*G609G/G609G*^ (G609G) (*n* = 20) and WT (*n* = 20) mice. Bars indicate mean ± standard error of mean and numbers above columns indicate *p* values. Differences were analyzed by unpaired *t* test. **D** Representative immunostaining of VSMCs from *Lmna*^*G609G/G609G*^ (G609G) and WT mice showing γH2Ax foci (green) and nuclei (blue). Scale bars, 10 µm. Graph shows mean of γH2Ax foci per nucleus in VSMCs from *Lmna*^*G609G/G609G*^ (G609G) (*n* = 71) and WT (*n* = 70) mice. Bars indicate mean ± standard error of mean and numbers above columns indicate *p* values. Differences were analyzed by unpaired *t* test. **E** Representative immunoblots showing AIF and cleaved Cas 3 protein levels in VSMCs from *Lmna*^*G609G/G609G*^ (G609G) and WT mice. Ponceau was used for normalization. **F** Immunostaining of VSMCs from *Lmna*^*G609G/G609G*^ (G609G) and WT mice showing cytochrome C (green) staining. Nuclei were stained with Hoechst (blue). Scale bars, 20 µm. **G** Immunostaining of *Lmna*^*G609G/G609G*^-VSMCs and WT-VSMCs showing AIF (green), mitochondria (red) and nuclei (blue). Upper scale bars, 10 µm and down scale bars, 5 µm. **H** Representative immunoblots showing Mfn1 an Mfn2 protein levels in VSMCs from *Lmna*^*G609G/G609G*^ (G609G) and WT mice. Ponceau was used for normalization. **I** Quantification of mitochondrial footprint (μm2) in VSMCs from *Lmna*^*G609G/G609G*^ (G609G) (*n* = 9) compared with WT (*n* = 10). Bars indicate mean ± standard error of mean and numbers above columns indicate *p* values. Difference was analyzed by unpaired *t* test. **J** Representative immunofluorescence micrographs of AIF staining in VSMCs from *Lmna*^*G609G/G609G*^ (G609G) (*n* = 9) and WT (*n* = 10) mice. The scan line graphs represent the intensity of AIF staining along the yellow lines. Bars indicate mean ± standard error of mean and numbers above columns indicate *p* values. Differences were analyzed by unpaired *t* test.

To determine the molecular cause of increased apoptosis, we next isolated VSMCs from *Lmna*^*G609G/G609G*^ mice (Fig. 2A). We first observed that mutated VSMCs displayed an increased cell death compared with VSMCs isolated from control mice (Fig. 2B). DNA damage as an end point of genotoxicity can be detected by “comet assay”. Comet assay performed in VSMCs from both control and *Lmna*^*G609G/G609G*^ mice showed longer tail lengths in mutant cells compared with controls, suggesting an increased level of DNA lesions (Fig. 2C). Consistently, an increased number of phosphorylated histone H2Ax (γH2Ax) foci, a highly sensitive biomarker of DNA damage and repair, was also observed in the nuclei of VSMCs from *Lmna*^*G609G/G609G*^ mice compared with controls cells (Fig. 2D). These DNA damage could suggest cellular apoptosis. Immunoblotting indicated that expression of AIF and cleaved-caspase 3 were increased in VSMCs from *Lmna*^*G609G/G609G*^ mice compared with controls (Fig. 2E). In agreement with what we observed on whole aorta, immunostaining experiments showed a massive increase in Cyt C expression in VSMCs from *Lmna*^*G609G/G609G*^ mice compared with controls (Fig. 2F). Given that apoptosis could be triggered by the release of AIF from the mitochondria into the cytosol and subsequent translocation to the nucleus, we analyzed AIF cellular localization. We found decreased localization of AIF in mitochondria in VSMCs from *Lmna*^*G609G/G609G*^ mice (Fig. 2G). Given that during apoptosis AIF is released from the mitochondrial intermembrane space to the cytosol, suggests that mitochondria may serve as a decision-maker regulating the viability of cells. We then studied the mitochondria network in VSMCs from *Lmna*^*G609G/G609G*^ mice. We showed that the expression of mitofusin-1, a known mediator of mitochondria fusion was reduced in mutated cells compared with control (Fig. 2H). This was associated with abnormal mitochondrial network in the mutated cells (Fig. 2I). Consistently, increased nuclear AIF localization in VSMCs from *Lmna*^*G609G/G609G*^ mice was detected by immunofluorescence microscopy (Fig. 2J). Collectively these results indicate that progerin expression causes increased cell death in cultured VSMCs.

### Altered poly(ADP-Ribosyl)ation causes loss of vascular smooth muscle cells in HGPS

**Fig. 3:**
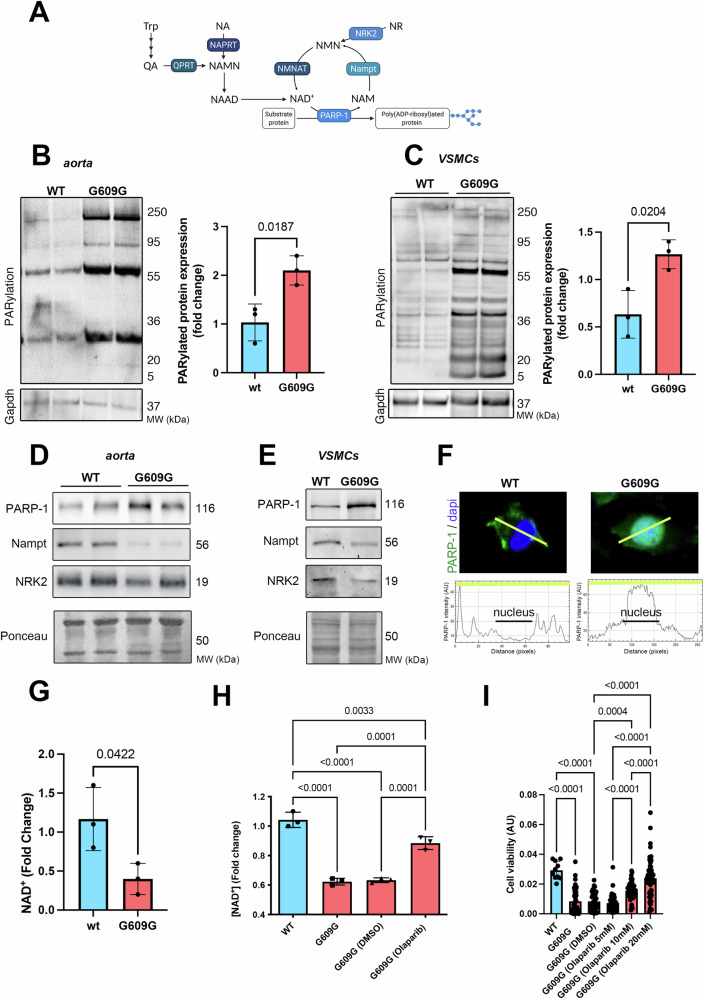
Increased global poly(ADP-Ribosylation) participates in altered NAD^+^ metabolism in *Lmna*^*G609G/G609G*^ models. **A** Schematic representation of the NAD^+^ biosynthesis. Trp tryptophan, NA quinolinic acid, QPRT quinolinic acid phosphoribosyltransferase, NAPRT nicotinic acid phosphoribosyltransferase, NMN nicotinamide mononucleotide, NAMN nicotinic acid mononucleotide, NAAD nicotinic acid adenine dinucleotide, NAM nicotinamide, NA nicotinic acid, NR nicotinamide riboside, NAD+ nicotinamide adenine dinucleotide, PARP-1 poly-ADP-ribose polymerase 1. **B** Representative immunoblot showing total poly(ADP-Ribosyl)ated protein level expression with PAR antibody (Fold change) in aorta from *Lmna*^*G609G/G609G*^ mice (*n* = 3) and WT mice (*n* = 3). Ponceau was shown as loading control. The bar graphs represent poly(ADP-Ribosyl)ated protein relative expression (mean ± standard error of means and numbers above columns indicate *p* values) (relative to ponceau). Difference was analyzed by unpaired *t* test. **C** Representative immunoblot showing total poly(ADP-Ribosyl)ated protein level expression with PAR antibody (fold change) in VSMCs from *Lmna*^*G609G/G609G*^ (G609G) (*n* = 3) and WT (*n* = 3) mice. Ponceau was shown as loading control. The bar graphs represent poly(ADP-Ribosyl)ated protein relative expression (mean ± standard error of means and numbers above columns indicate *p* values) (relative to ponceau). Difference was analyzed by unpaired *t* test. **D** Representative immunoblots showing PARP-1, Nampt and NRK2 protein level in aorta from *Lmna*^*G609G/G609G*^ mice (*n* = 3) and WT (*n* = 3) mice. Ponceau was shown as loading control. **E** Representative immunoblots showing PARP-1, Nampt and NRK2 protein level in VSMCs from *Lmna*^*G609G/G609G*^ (G609G) (*n* = 3) and WT (*n* = 3) mice. Ponceau was shown as loading control. **F** Representative immunofluorescence micrographs of PARP-1 staining in VSMCs from *Lmna*^*G609G/G609G*^ (G609G) and WT mice. The scan line graphs represent the intensity of PARP-1 staining along the yellow lines. **G** Quantification of NAD^+^ content (fold change) in VSMCs from *Lmna*^*G609G/G609G*^ (G609G) (*n* = 3) and WT (*n* = 3) mice. Bars indicate mean ± standard error of mean and numbers above columns indicate *p* values. Difference was analyzed by unpaired *t* test. **H** Quantification of NAD^+^ content (fold change, NAD^+,^ and NADH) in VSMCs from WT (*n* = 3), and *Lmna*^*G609G/G609G*^ (G609G) mice (*n* = 3) treated (*n* = 3) or not (*n* = 3) with Olaparib (5 mM). Bars indicate mean ± standard error of mean and numbers above columns indicate *p* values. Difference was analyzed by one-way ANOVA followed by Tukey’s multiple comparison test. **I** Assessment of cell viability of VSMCs from *Lmna*^*G609G/G609G*^ (G609G) mice treated or not with Olaparib (5, 10, and 20 mM) (*n* = 10 bright fields from five independent plates per condition (pooled)). Bars indicate mean ± standard error of mean and numbers above columns indicate *p* values. Each dot represents the quantification for one microscopy image. Difference was analyzed by one-way ANOVA followed by Tukey’s multiple comparison test.

As accumulation of DNA breaks and increased apoptosis were observed in VSMCs expressing progerin, we next aimed to investigate the role of poly(ADP-Ribosyl)ation, a post-translational modification involved in DNA damage repair in the presence of NAD^+^ [18] (Fig. 3A). Immunoblotting using poly(ADP-ribose) (PAR) antibody showed a significantly increased poly(ADP-Ribosyl)ation in aorta from *Lmna*^*G609G/G609G*^ mice (Fig. 3B), as well as in cultured VSMCs from *Lmna*^*G609G/G609G*^ mice compared with controls (Fig. 3C). Poly(ADP-Ribosyl)ation is catalyzed by PARPs enzymes, among which PARP-1 is the main isoform in the nucleus [19]. We found that progerin expression correlates with increased PARP-1 expression compared with control in whole aorta (Fig. 3D) as well as in isolated VSMCs from *Lmna*^*G609G/G609G*^ mice (Fig. 3E). We found that PARP-1 was predominantly nuclear in isolated VSMCs from *Lmna*^*G609G/G609G*^ mice compared with controls (Fig. 3F). PARP-1 is a DNA damage sensor playing a critical role in the DNA repair pathway. PARP-1 uses NAD^+^ as a substrate, and this consumption of NAD^+^ by damage-induced PARP activation was expected to hinder cellular energy metabolism [20]. Given that the major pathway for maintaining intracellular NAD^+^ levels is the salvage pathway, we assessed the expression of Nampt and NRK2, two enzymes that are important building blocks for the production of NAD^+^ [17]. Immunoblot analysis revealed a decreased expression of Nampt and NRK2 in aorta from *Lmna*^*G609G/G609G*^ mice (Fig. 3D) as well as in VSMCs from *Lmna*^*G609G/G609G*^ mice compared with controls (Fig. 3E). Lastly, quantification of the steady-state NAD^+^ level in isolated VSMCs showed a significantly reduced NAD^+^ content in VSMCs from *Lmna*^*G609G/G609G*^ mice compared with controls (Fig. 3G). Altogether, these data show that the presence of progerin correlates with decrease NAD^+^ content in VSMCs and suggest that NAD^+^ depletion could participate in increased VSMCs loss in aorta from HGPS. Given that PARP-1 uses NAD^+^ as a substrate to ADP(ribosyl)ate various target proteins, we next assessed the impact of inhibiting PARP-1 (using Olaparib) on NAD^+^ level in isolated VSMCs. Our data showed that Olaparib treatment increased the NAD^+^ cellular content in cultured VSMCs from *Lmna*^*G609G/G609G*^ mice compared with controls (Fig. 3H). We then addressed the role of PARP-1 inhibition on the cell death observed in mutated VSMCs. We observed that mutated VSMCs treated with Olaparib displayed an increased cell viability compared with untreated VSMCs isolated from *Lmna*^*G609G/G609G*^ mice, in a dose-dependent manner (Fig. 3I). Collectively these results indicate that increased PARP-1 expression in VSMCs expressing progerin participated in the NAD^+^ cellular content and the cell death.

### Identification of a novel compound that inhibits PARP-1 activity and increases NAD^+^ levels in vascular smooth muscle cells expressing progerin

**Fig. 4:**
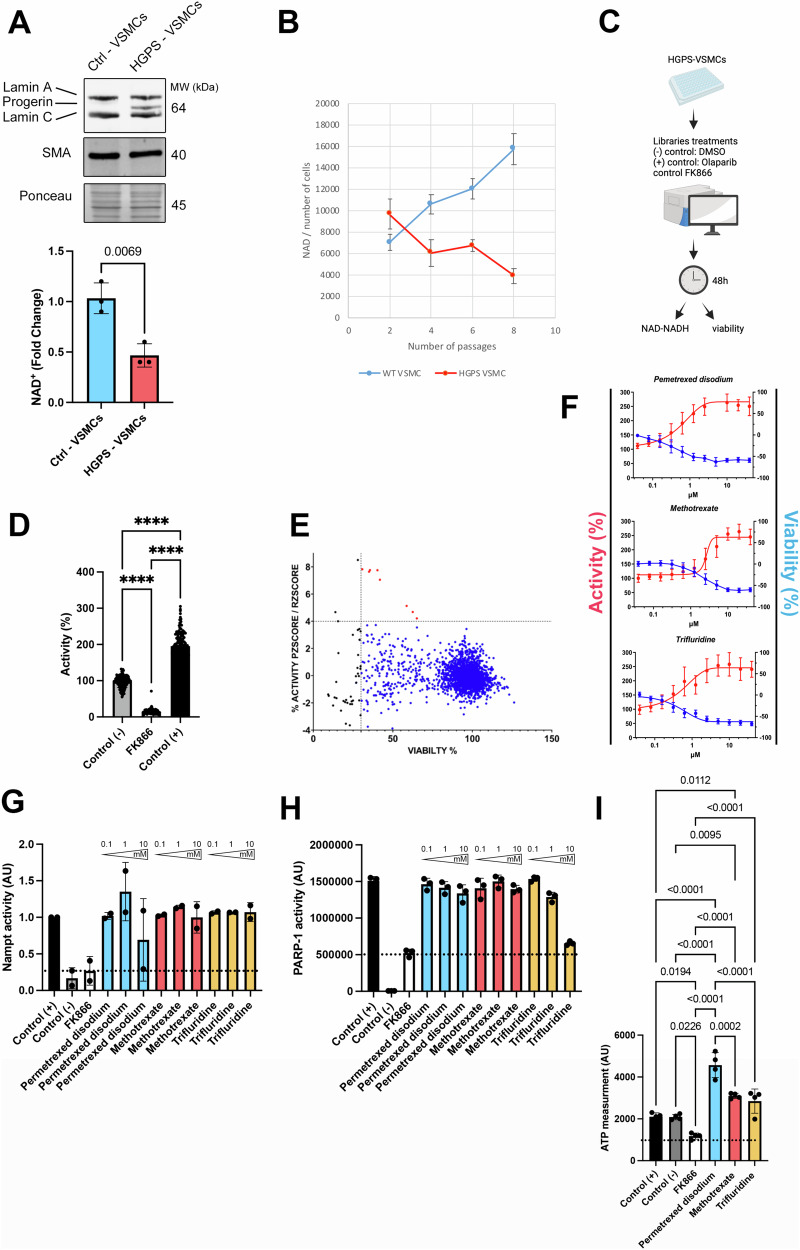
High-throughput screening on iPSC derived VSMCs from HGPS patients allows trifluridine identification for treatment of *Lmna*^*G609G/G609G*^ model. **A** Representative immunoblots showing lamin A, progerin, lamin C and SMA protein levels in isolated VSMCs derived from HGPS iPSCs patient (HGPS-VSMCs). Ponceau was used for normalization. Immunoblots are associated to a quantification of NAD^+^ content (fold change, NAD^+^ and NADH) in HGPS-VSMCs (*n* = 3) and control-VSMCs (*n* = 3). Bars indicate mean ± standard error of mean and numbers above columns indicate *p* values. Differences were analyzed by unpaired *t* test. **B** Graph shows NAD^+^ level (NAD^+^ and NADH) normalized by the quantity of cells during the different cell passages (*n* = 3). Bars indicate mean ± standard error of mean. **C** Scheme of the high-throughput screening performed in iPSC derived VSMCs from HGPS patients. **D** Activity (NAD^+^ and NADH) normalized by the cell number expresses in % in DMSO- (control−), FK866− or Olaparib-(control+) treated iPSC derived VSMCs from HGPS patients. Bars indicate mean ± standard error of mean of 16 replicates wells in four independent plates (pooled). **E** Primary screen cell-based assay. Dot plot representation of the effects of the library compounds on NAD^*+*^ (NAD^+^ and NADH), normalized to negative control and cell viability. **F** Graphs show dose-response effect of three validated compounds with the activity (red) (NAD^+^ level normalized by the quantity of cells) and the viability (blue) compared to DMSO treatment. **G** NAMPT activity after different concentrations (0.1, 1, 10 mM) of pemetrexed disodium, methotrexate or trifluridine treatment (*n* = 3 for each condition). Bars indicate mean ± standard error of mean and numbers above columns indicate *p* values. Differences were analyzed by one-way ANOVA. **H** PARP-1 activity after different concentrations (0.1, 1, 10 mM) of pemetrexed disodium, methotrexate or trifluridine treatment (*n* = 3 for each condition). Bars indicate mean ± standard error of mean and numbers above columns indicate *p* values. Differences were analyzed by one-way ANOVA followed by Tukey’s multiple comparison test. **I** ATP measurement activity per cell after 1 mM of pemetrexed disodium, methotrexate or trifluridine treatment (*n* = 3 for each condition). Bars indicate mean ± standard error of mean and numbers above columns indicate *p* values. Differences were analyzed by one-way ANOVA followed by Tukey’s multiple comparison test.

iPSCs enable the generation of previously unattainable, scalable quantities of disease-relevant tissues from patients suffering from essentially any genetic disorder. This cellular material has proven instrumental for drug screening efforts on these disorders, and has facilitated the identification of novel therapeutics for patients. Accordingly, in this study, we then used an iPSC-based disease model of HGPS to consolidate our findings in human and identify drug candidates increasing NAD^+^ content in VSMCs. We used VSMC differentiated from two controls and two HGPS iPSC lines, previously reported [21]. We confirmed a significant decreased level of NAD^+^ in VSMCs derived from HGPS iPSCs (HGPS VSMCs) compared with control cells, consistent with our results in VSMCs from *Lmna*^*G609G/G609G*^ mice (Fig. 4A). More importantly, our results revealed that this difference in NAD^+^ between HGPS VSMCs and control cells was increased along passages in culture (Fig. 4B). We then focused on the identification of therapeutic compounds able to increase NAD^+^ content in HGPS VSMCs. We developed a high throughput-screening assay in 384-well plates that allow to quantify both NAD^+^ cellular level and cell viability, following drug treatment (Fig. 4C). Specificity of the screening assay was carried out using 48 h treatments with positive and negative controls. On one hand, treatment with FK866, a highly specific non-competitive inhibitor of NAMPT was used to reduce NAD^+^ content and on the other hand treatment with Olaparib, a potent PARP-1/2 inhibitor was used to increase NAD^+^ content (Fig. 4D). Robustness of the assay was evaluated through the calculation of a Z’ factor superior to 0.5 between positive and negative controls, in four independent plates (Fig. 4D). We next used this assay to perform a high-throughput screening of a library of 2237 drugs at 5 µM on HGPS VSMCs (Fig. 4E). Quality control of the screening procedure was performed by measuring a Z’ factor between positive and negative controls in the screening plate superior to 0.5. Hits were considered as potential candidates when their effect was greater than three standard deviations from the mean of all tested compounds following both plate and run Z score analysis and without affecting cell viability to an extent of greater than 30%. This led to a first list of 8 drug candidates (Table S1). Following retest and dose-response experiments, 3 compounds, pemetrexed disodium, methotrexate and trifluridine, were validated with EC50 of 0.6 µM, 3 µM and 0.44 µM, respectively (Table S1 and Fig. 4F). We next investigated the mechanism of action of those drugs by measuring their effect on NAMPT and PARP-1 enzymatic activity, using biochemical procedure. Although our results revealed that these drugs were acting on NAD^+^ content through an increase of NAMPT activity at any given doses (Fig. 4G), measure of PARP-1 activity revealed a direct and dose dependent inhibition by trifluridine (Fig. 4H). Given that NAD^+^ is important for the mitochondrial OXPHOS function, we next assessed the ATP production. The assay confirmed that treatment with these 3 drugs led to a significant increase of ATP production in HGPS VSMCs (Fig. 4I). We showed that trifluridine has no impact on proliferation, of HGPS VSMCs as measure by Ki67 staining (Fig. S3). Together, these results identify trifluridine as a potential drug candidate to increase NAD^+^ content in HGPS VSMCs through PARP-1 inhibition.

### TAS-102 restores NAD^+^ levels in vascular smooth muscle cells and improves aortic disease in HGPS

**Fig. 5:**
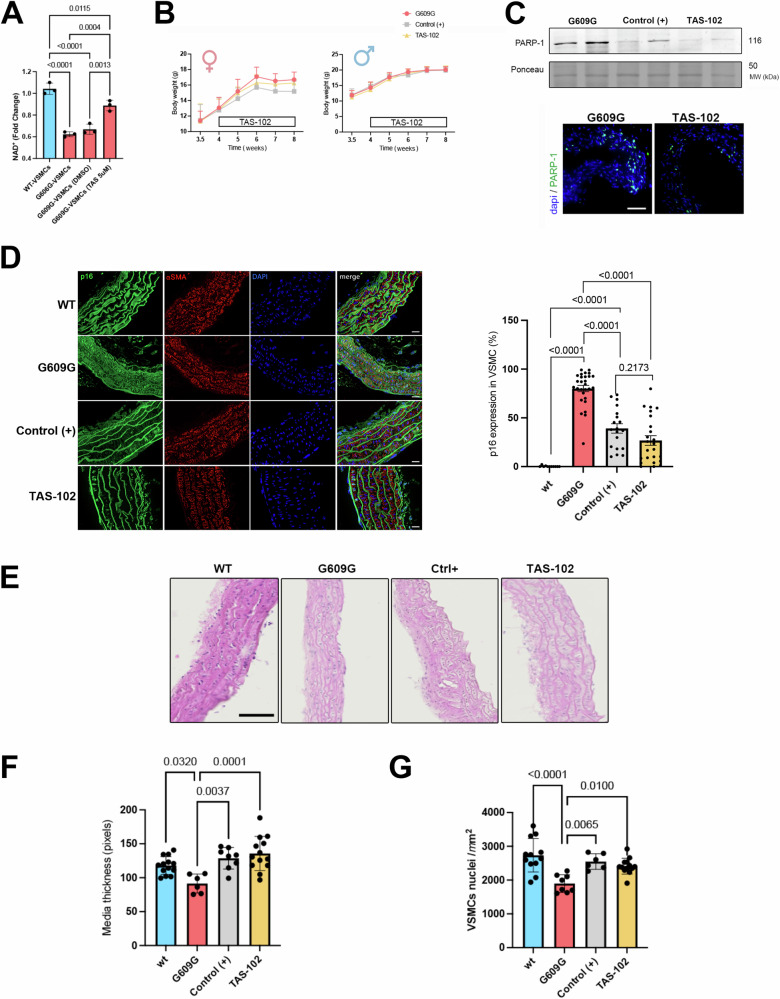
TAS-102 treatment restores NAD^+^ levels and improves aortic disease. **A** Quantification of NAD^+^ content (fold change) in VSMCs from WT and *Lmna*^*G609G/G609G*^ (G609G) mice treated with TAS-102 (5 µM) or DMSO (n = 3 per condition). Bars indicate mean ± standard error of mean and numbers above columns indicate *p* values. Differences were analyzed by one-way ANOVA followed by Tukey’s multiple comparison test. **B** Graph of body weight of mice throughout 4 weeks of treatment with TAS-102 (*n* = 10), Olaparib “Control (+)” (*n* = 10) or DMSO (*n* = 10). **C** Top: Representative immunoblots showing PARP-1, expression in the aorta of *Lmna*^*G609G/G609G*^ mice treated with Olaparib “Control (+)” and with TAS-102. Ponceau was shown as loading control. Bottom: Immunostaining of the aorta (the media) from *Lmna*^*G609G/G609G*^ mice treated or not with TAS-102 showing PARP-1 (green) and nuclei (blue). Scale bars, 50 µm. **D** Immunostaining of aorta cross sections from *Lmna*^*G609G/G609G*^ (G609G) and WT mice showing α-SMA (red) and p16 (green) showing senescent cells. Nuclei stained with DAPI (blue). Scale bar, 20 µm. Graph compares the percentage of p16 senescent VSMCs in aortic arch quantification in *Lmna*^*G609G/G609G*^ (G609G) (*n* = 6) and *Lmna*^*G609G/G609G*^ (G609G) treated with TAS-102 (*n* = 6), Olaparib (*n* = 5) compared with WT (*n* = 3) mice. Bars indicate mean ± standard error of mean and number above columns indicate *p* values. Differences were analyzed by one-way ANOVA test followed by Tukey’s multiple comparison test. **E** Representative aorta cross sections from *Lmna*^*G609G/G609G*^, WT and *Lmna*^*G609G/G609G*^ mice treated with either TAS-102, Olaparib “Control (+)” or DMSO (Control). Scale bar, 50 µm. **F** Media thickness quantification in *Lmna*^*G609G/G609G*^ mice treated with TAS-102, Olaparib “Control (+)”, DMSO and in WT (*n* = 4 per condition). Quantification performed on different regions of the media (each represented by a dot on the graph). Differences were analyzed by one-way ANOVA followed by Tukey’s multiple comparison test. **G** Number of VSMCs evaluated by nuclei count in *Lmna*^*G609G/G609G*^ mice treated with TAS-102, Olaparib “Control (+)”, DMSO and in WT (*n* = 4 per condition). Quantification performed on different regions of the media (each represented by a dot on the graph). Differences were analyzed by one-way ANOVA.

As a last step, we undertook to evaluate the effect of trifluridine on loss of VSMCs in vivo using *Lmna*^*G609G/G609G*^ mice. When taken orally, trifluridine is rapidly degraded in the liver into inactive metabolites via the enzyme thymidine phosphorylase [22, 23]. To overcome this limit, it is usually administered in combination with the thymidine phosphorylase inhibitor tipiracil. We showed that both trifluridine alone or in combination with tipiracil (TAS-102) have similar benefit on NAD^+^ and ATP cellular content on HGPS VSMCs (Fig. S4). All further experiments were then performed using trifluridine/tipiracil (TAS-102) combination. The goal of this approach is to test if restoring NAD^+^ level could impact positively aortic structure and function. We first evaluated the effect of TAS-102 on NAD^+^ level in primary cultured VSMCs from *Lmna*^*G609G/G609G*^ mice. After 48 h of treatment, TAS-102 (5 µM) was able to significantly increase NAD^+^ level in mutant VSMCs compared with DMSO-treated cells (Fig. 5A).

Considering the important role of NAD^+^ in the development of vascular disease in HGPS, we next tested the benefit of TAS-102 in vivo. We then treated the homozygous *Lmna*^*G609G/G609G*^ mice to study the benefit of TAS-102 on progeroid symptoms, due to the severe manifestation of the disease in this model. Four-week-old *Lmna*^*G609G/G60G*^ mice were treated orally for a period of 4 weeks with TAS-102, with Olaparib (a reference PARP-1 inhibitor molecule) or with DMSO. At the end, all mice were alive and no sign of pain was observed. Mutant mice treated with TAS-102 displayed no significant increased body weight compared with mice treated with Olaparib or DMSO (Fig. 5B). We treated *Lmna*^*G609G/G609G*^ mice with 150 mg/kg of TAS-102. Given that TAS-102 is a potent PARP-1 inhibitor, we assessed PARP-1 expression in whole aorta from treated-*Lmna*^*G609G/G609G*^ mice. We showed a decreased PARP-1 expression in treated-*Lmna*^*G609G/G609G*^ mice compared with untreated mice (Fig. 5C). The *Lmna*^*G609G/G60G*^ mice exhibit important cardiovascular alterations with loss of VSMCs in the aortic arch causing media layer thinning in particular in the aortic arch [12]. *Lmna*^*G609G/G60G*^ mice treated with TAS-102 or Olaparib showed a significantly reduced senescence (Fig. 5D) as well as a significantly increased media thickness compared with vehicle-treated (Fig. 5E). The media thickness in the treated mice is then comparable with the control mice (Fig. 5F). This is associated with a significant increased number of VSMCs in the treated mice compared with vehicle-treated animals (Figs. 5G and S5). Collectively, these results indicate that TAS-102 promotes a healthier aortic phenotype in *Lmna*^*G609G/G60G*^ mice.

## Discussion

**Fig. 6:**
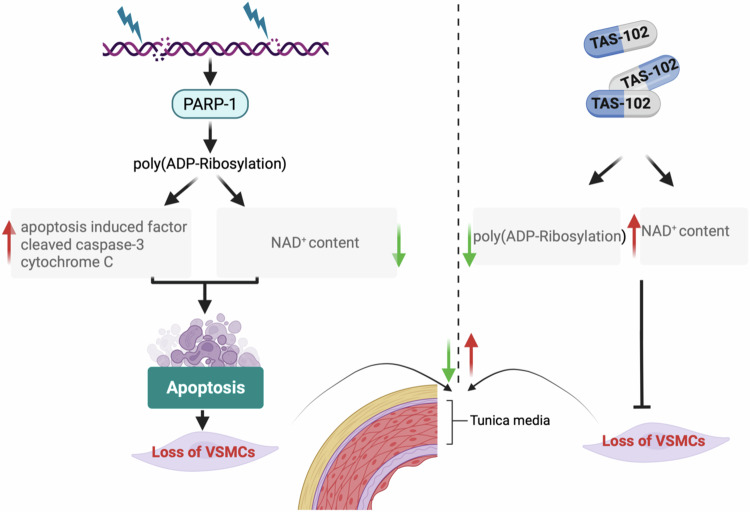
Schematic representation of the PARP-dependent cell death program activation contributing to the death of aortic VSMCs in HGPS and beneficial effects of TAS-102 treatment. Created with BioRender.com.

Decreased VSMCs abundance and functionality causing altered aortic integrity is a hallmark of HGPS [24, 25]. Our results provide new insight into the underlying mechanism with the first demonstration of a link between the accumulation of progerin and altered poly(ADP-Ribosyl)ation in VSMCs from *Lmna*^*G609G/G60G9*^ mice (Fig. 6). This poly(ADP-Ribosyl)ation defect is associated with reduced NAD^+^ level and DNA damage accumulation. These defects cause PARP-dependent cell death program activation likely contributing to the death of aortic VSMCs observed in *Lmna*^*G609G/G60G9*^ mice. Treatment of *Lmna*^*G609G/G609G*^ mice with PARP-1 inhibitors activity led to a significant improvement of their aortic phenotype (Fig. 6).

NAD^+^ level plays a major role in the regulation of many biological processes [26]. NAD^+^ is constantly degraded by consumer enzymes and synthesized by different pathways, to maintain homeostasis [26]. Age-related NAD^+^ decline has been reported in many study models such as yeast, mice as well as humans [27–29], and contributes to altered homeostasis and increased disease vulnerability [30]. In mice, decreased NAD^+^ content has been associated with hallmarks of aging such as mitochondrial defect, inflammation, DNA damage, signaling alterations, chromatin and epigenetics changes [31]. NAD^+^ has therefore emerged as a potential treatment targeting age-related disease [32]. We speculated that NAD^+^ depletion in VSMCs from *Lmna*^*G609G/G609G*^ mice may play a similar role to NAD^+^ in physiological aging. It has been previously shown in VSMCs differentiated from iPSCs derived from HGPS fibroblasts, that decreased PARP-1 activity leads to aberrant activation of non-homologous end-joining repair resulting in cellular growth arrest leading to mitotic catastrophe and cell death [33]. These results are not correlated to our findings. However, we confirmed these results in primary VSMCs from *Lmna*^*G609G/G609G*^ mice, emphasizing the truthfulness of our findings. Given that PARP-1 activity is linked to NAD^+^ cellular content, these works pinpointed the key role for NAD^+^ depletion in the loss of VMSCs in *Lmna*^*G609G/G609G*^ mice. Aortic wall degeneration in *Nampt* knock-out mice was associated with decreased NAD^+^ levels and increased DNA damage in VSMCs [34]. We have here reported a decreased *Nampt* expression and NAD^+^ drop in VSMCs from *Lmna*^*G609G/G609G*^ mice, associated with an increase in DNA damages. These results suggest that progerin expression in VSMCs leads to persistent DNA damage response by poly(ADP-Ribosyl)ation. This is supported by other studies showing that the accumulation of prelamin A in VSMCs causes an increased levels of DNA damage [35] and promotes VSMC osteogenic differentiation and mineralization [36]. It is now well-recognized that cells under genotoxic stress activates PARP-1 overactivation to repair DNA damage, decreased NAD^+^ levels, leading to cell apoptosis [37]. To confirm this, treatment with PARP inhibition on DNA damages is required.

The rescue of NAD^+^ cellular content by targeting PARP-1 activity in *Lmna* mutated VSMCs is an interesting approach to normalize VSMCs homeostasis. In a mouse model of Cockayne syndrome, another premature aging syndrome, treatment with PARP-1 inhibitors promoted lifespan and decreased aging associated phenotypes [38]. These data suggest that PARP-1 could be a relevant target for the treatment of progeria. In this context, we used high-throughput screening to identify a potential compound for the treatment of *Lmna*^*G609G/G609G*^ mice. We identified trifluridine as a beneficial molecule to restore NAD^+^ levels through decrease of PARP-1 activity in VSMCs from *Lmna*^*G609G/G609G*^ mice. Moreover, we demonstrated that TAS-102 treatment alleviates aortic VSMCs loss, collagen deposition, and bone thickness defect in *Lmna*^*G609G/G609G*^ mice, confirming the direct link between PARylation defect and progeria phenotype. Currently, many potential therapeutical strategies for progeria have emerged based on inhibition of progerin expression, inhibition of progerin prenylation, increased clearance of progerin or decreased toxic effects of progerin [39]. Also, genome and base editing approaches have been successfully developed in vivo [40–42]. Given that progerin toxicity is induced by the farnesyl group, several molecules exist to inhibit the prenylation of progerin and in November 2020, lonafarnib received its first approval in the United States to reduce the risk of mortality in HGPS [43]. HGPS patients treated with lonafarnib three times a day for 30 days had an increase in longevity of ~2.2 years compared to untreated patients [44]. Although many therapeutic strategies are still under investigation, it is now well-recognized that combination of therapies can have beneficial potentiating effects. Our study shows that TAS-102, targeting poly(ADP-Ribosyl)ation, could be a treatment to improve vascular pathology in progeria. NAD^+^ boosting strategies have emerged to treat cardiovascular diseases [45, 46]. Our study was focused on the preservation of aortic VSMCs in progeria mouse model. To move further with pre-clinical development of TAS-102, survival studies of treated mice are necessary. It is of interest to note that TAS-102 has been approved by US Food and Drug Administration for metastatic colorectal cancer. Hence, TAS-102 represents a manageable and effective therapeutic opportunity and appeared to be well tolerated with generally mild side effects. Moreover PARP-1 inhibitors such as Olaparib are commonly used in cancer treatment [37], confirming that PARP-1 inhibitors are good candidate for the treatment of diseases. Positive results after *Lmna*^*G609G/G609G*^ mice treated mice with TAS-102, will allow development of treatments to reduce cardiovascular pathology in progeria or in aging in general.

In conclusion, this work aimed to address important issues that severely hinder the understanding of premature ageing syndromes. We highlighted the role of poly(ADP-Ribosyl)ation in loss of VSMCs in the aorta of a HGPS mouse model, resulting in development of the cardiovascular pathology. We identified a novel compound, TAS-102, which counteracts the increase of poly(ADP-Ribosyl)ation and allows the maintenance of VSMCs in aorta. Therefore, poly(ADP-Ribosyl)ation has emerged as an important target for the development of treatment to impede the cardiovascular pathology in HGPS patients.

## Materials and methods

### Mouse model

Knock-in mice model *Lmna*^*G609G/G609G*^ carrying the *Lmna* c.1827C > T (p.Gly609Gly) mutation have been previously described [12]. Mice were fed chow and housed in a disease-free barrier facility with 12 h light/12 h dark cycles. All animal studies were approved by the French Ministry of Health at the Marseille medical genetics institute for the Care and Use of Experimental Animals [2019070912126767-V2 #21407].

### Treatment

The mouse model generation and phenotyping services were provided by PHENOMIN—iCS (www.phenomin.fr). 15 *Lmna*^*G609G/G609G*^ females and 15 homozygous *Lmna*^*G609G/G609G*^ males were used for the study. At the age of 4 weeks, mice were randomized, based on their body weights, into three groups (5 males and 5 females per group), and treated orally for a period of 4 weeks as follows: (1) mutant males (*n* = 5) + mutant females (*n* = 5) treated with the TAS-102 at a dose of 150 mg/kg/day by oral gavage; (2) mutant males (*n* = 5) + mutant females (*n* = 5) treated with Olaparib at a dose of 100 mg/kg/day by oral gavage; (3): 5 mutant males + 5 mutant females treated with vehicle (corn oil 80% / DMSO 20%) by oral gavage. All animal studies were approved by the French Ministry of Health at the Marseille medical genetics institute for the Care and Use of Experimental Animals [2019070912126767-V2 #21407, 2021011214302716-V2 #28933].

### VSMCs isolation and culture

Primary VSMCs were prepared from aorta of 4 weeks-old WT and *Lmna*^*G609G/G609G*^ mice following a protocol adapted from [47]. After asphyxiate mouse with CO_2_ for 2 min, heart was perfused through the apex with 1 ml 1× sterile HBSS (Gibco). After dissection, 2 aortas from 2 mice of the same littermate were pooled into the HBSS in 15 ml conical in a cool place. Aortas were incubated at 37 °C for 8–10 min with enzyme solution containing collagenase (1 mg/ml) (Gibco), soybean trypsin inhibitor (1 mg/ml) (Gibco), elastase (0.744 units/ml) (Gibco), 1% pen/strep (Gibco) and HBSS. Aortas were washed with DMEM/F12 (Gibco) media before removing endothelial cell layer. Aortas were opened longitudinally to remove any blood clots and endothelial cell layer by gentle scrapping the inside of the vessel and incubated at 37 °C in 5% CO_2_ in the incubator for about an hour within enzyme solution. After digestion, the digested tissue was triturate by shredding, wash with DMEM/F12 media, and centrifuge 3 times. After centrifugation, cells were seeded on 2 wells of a 24 wells dish DMEM/F12 media. VSMCs were then cultured in DMEM/F12 media containing 10% fetal bovine serum (Gibco) and 1% pen/strep. Cell viability was assessed by reporting the number of VSMCs per μm^2^ by immunofluorescence microscopy (Dapi staining).

### Fibroblast culture and reprogramming

HGPS Fibroblasts (8243) used in this study was isolated from a patient biopsy taken in the Assistance Publique Hôpitaux de Marseille during diagnostic procedures referred as HGPS-VSMCs in this study as previously described [48]. Informed consents were obtained from the parents of the patient included in this study, complying with the ethical guidelines of the institutions involved and with the legislation requirements of the French ministry. The HGPS cell line explored in this study have been prepared and stored according to the French regulation by the labeled Biological Resource Center of Tissues, DNA, Cells (CRB TAC), Department of Medical Genetics, la Timone Hospital, Marseille (Dr A De Sandre-Giovannoli and Mrs K. Bertaux). The fibroblast cell line used belong to a biological sample collection declared to the French ministry of Health whose use for research purposes was authorized by the French ministry of Health. Control cell lines were used in this study. Cell line (4603) was provided by Coriell Cell Repository (Camden, USA) (referred as WT VSMCs in this study). Cells were obtained from the NINDS Human Genetics Resource Center DNA and Cell Line Repository. NINDS Repository sample numbers corresponding to the samples used are GM4603. Cultures were maintained in Dulbecco’s modified Eagle’s medium + GlutaMAX II + 4500 mg/L D-Glucose (Gibco), supplemented with 20% fetal bovine serum (research grade, Sigma) and 1% sodium pyruvate 100 mM (Life technologies). Cell cultures were maintained at 37 °C, in 5% CO_2_ in a humidified atmosphere, with the media changed every 2 days. Fibroblasts from the four cell lines were reprogrammed into iPS cells using Yamanaka’s original method with OCT4, KLF4, SOX2, c-myc, and transferred using retroviral vectors.

### Generation of disease-specific induced pluripotent stem cells

As previously described in [48], WT and HGPS iPSCs were grown in colonies on mouse embryonic fibroblasts, inactivated with 10 mg/ml mitomycin C seeded at 30,000 cells/cm^2^, and grown as previously described. The authenticity of the human iPSCs was confirmed with the expression of a panel of pluripotent markers.

### In vitro differentiation in VSMCs

SMC differentiation of iPSC was performed using embryoid body (EB) formation. EBs were cultured for 10 days at 37 °C, in 5% CO_2_ in a humidified atmosphere, with media changes every 2 days. CD34^+^ cells were isolated from EBs on day 10. The percentage of CD34^+^ cells in EBs was between 0.4% and 1.5%. Isolated cells were grown on 24-well plates (~30,000 cells/cm^2^) coated with 0.1% gelatin, in the presence of endothelial growth medium-2 (EGM-2, Lonza) supplemented with PDGFBB (50 ng/mL, Prepotech). After 4 passages, the medium was replaced with Smooth Muscle Growth Medium-2 (SmGM-2) (Lonza CC-3182) for 4 additional passages. Cell cultures were maintained at 37 °C, in 5% CO_2_ in a humidified atmosphere, with the media changed every 2 days.

### Immunoblotting

For protein extraction, tissues or whole-cell lysates were added in FastPrep tubes (MP Biomedicals) with 300 µl of lysis buffer (Cell Signaling). After five FastPrep cycles, samples were centrifuged and the supernatant were then collected. The lysates were separated by 10% SDS-polyacrylamide gel electrophoresis and transferred to nitrocellulose membranes (Invitrogen). Blotted membranes being were blocked in 5% bovine serum albumin (BSA) in Tris–buffered saline containing 1% Tween 20 (TBS-T) for 1 h at room temperature followed by incubation overnight at 4 °C with the appropriate primary antibody (Table S2). After washing with TBS-T, membranes were for 1 h at room temperature incubated with secondary anti-rabbit or anti-mouse antibodies conjugated to HRP. After washing with TBS-T the chemiluminescent signal was revealed using ChemiDoc MP Imaging System (Biorad). Bands were quantified using Image J software.

### Immunofluorescence and histology

Tissues were embedded in Tragacanth (Sigma) before being frozen and sectioned (10 µm) with cryostat (Leica) for staining. Sections or cells were fixed in 4% formaldehyde (Electron Microscopy Sciences) stained with Hematoxylin & Eosin (H&E) (Biogents). Tissues from treated animals were embedded in paraffin and sectioned at 5–7 µm of thickness with microtome (Leica). H&E staining (Sigma, Microm Microtech) was performed on aortas and hearts and Sirius Red staining (Sigma) was performed on hearts. Sections were imaged with Scanner Leica at magnification 20× with Zen software. For immunostaining, samples were incubated in blocking solution containing (5% Goat Serum + 1%BSA + 0.2% Triton). Primary antibodies (Table S2) were incubated overnight at 4 °C in blocking solution. After washing with PBS (Gibco), samples were incubated with secondary antibodies and Hoechst (Invitrogen) for 1 h at room temperature. Slices were mounted in Fluorescence Mounting Medium (Dako). Immunofluorescence microscopy was performed using an Axiophot microscope (Carl Zeiss). Most of the images were digitally deconvolved using Autodeblur v9.1 (Autoquant) deconvolution software and processed using Adobe Photoshop 6.0 (Adobe Systems). The microscope was controlled by MetaMorph 7.10 software (Molecular Devices) with a pixel resolution of 0.11 µm/px at 16-bit. The images were processed by FIJI/ImageJ software. Analysis of mitochondrial morphology was performed using the macro MiNA in Fiji software (ImageJ), which allows measurement of mitochondrial footprints (mitochondrial coverage area).

### TUNEL assay

TUNEL staining were performed using a TUNEL cell detection kit (Roche Molecular Biochemicals, Mannheim, Germany) according to the manufacturer’s protocol. Aorta cross sections were exposed to a medium containing 125 μM neomycin and 500 μM sodium selenite for 1 h. Slices were then washed with PBS and fixed in 4% paraformaldehyde before being incubated with 50 μL of TUNEL reaction mixture (TdT and fluorescein–dUTP) at 37 °C for 60 min in a humid atmosphere.

### NAD^+^ concentration quantification

The concentration of NAD^+^ was determined using a spectrophotometric assay as described previously [49]. Absorption measurements were carried out in 96-well plates by spectrophotometry (Tecan).

### Comet assay

Comet assay was performed according to instructions provided with the OxiSelect Comet Assay Kit (Cell Biolabs). Cells were lysed for 30 min at 4 °C, immersed in 1× TBE buffer for 15 min, and electrophoresis was performed at neutral pH at 1 V/cm (measured electrode to electrode) for 30 min. Slides were immersed in H_2_O for 5 min, 70% ethanol for 5 min, and dried at room temperature overnight. Samples were then stained with SYBR Green and imaged with a confocal microscope. DNA damage was assessed by measuring comet tail length using OpenComet software (v1.3.1).

### VSMCs treatment

VSMCs were seeded and treated 12 h after seeding with 0.1% dimethyl sulphoxide and 5 µM of compounds added to DMEM/F12 (Gibco). Treatment was repeated 24 h after the initial treatment. Cells were analyzed after 48 h of treatment.

### Measurement of PARP-1 activity

Measure of PARP-1 was performed following the PARP-1 Colorimetric Assay Kit (BPS Biosciences). According to the manufacturer’s protocol, 50 μl of histone solution was added to each well of a 96-well plate and incubate at 4 °C overnight. After 3 PBST buffer washes, the liquid excess was removed onto clean paper towel. Each well was blocked at room temperature for 60–90 min with blocking buffer to every well. After 3 PBST buffer washes, the liquid excess was removed onto clean paper towel. The mixture containing PARP buffer, PARP assay and activated DNA was prepared according to the manufacturer’s protocol and 25 µl were added to every well. Five microlitres of Inhibitor solution were added for “test Inhibitor” and 5 µl of the same solution without inhibitor were added for the “positive control” and Pemetrexed disodium, Methotrexate and trifluridine were added in other well in triplicates. Reaction was initiated by adding 20 μl of diluted PARP-1 enzyme to the wells designated and incubated at room temperature for 1 h. Reaction mixture after 1 h was discarded and plate was three times washed with PBST. Fifty microlitres of diluted Streptavidin-HRP were added to each well for 30 min at room temperature. After 3 washes with PBST, 100 μl of the colorimetric HRP substrate was added to each well and the plate was incubated at the room temperature until blue color is developed in the positive control well (about 15 ~ 20 min) to fully develop the color. The absorbance was measured at 450 nm using UV/Vis spectrophotometer microplate reader (BMG Labteck).

### Measurement of NAMPT activity

Measure of NAMPT activity was performed following the Colorimetric Assay Kit (Abcam), according to the manufacturer protocol. 60 μl of Nampt reaction solution was added to every well and the absorbance was measured at 450 nm using UV/Vis spectrophotometer microplate reader (BMG Labteck).

### Cell viability quantification

Live VSMCs were stained with calcein (Thermo Fisher Scientific) and dead cells were labeled with the ethidium homodimer dye (Thermo Fisher Scientific). Cells were viewed under a light confocal microscope. Images were processed and cells viability was calculated using FIJI/ImageJ software.

### β-galactosidase staining

β-galactosidase staining was performed following the Senescence Detection Kit protocol (Sigma-Aldrich). The assay is based on a histochemical stain for senescence-associated β-galactosidase (β-gal) activity at pH 6. Cells were washed in 1× PBS and incubated with fixation buffer 7 min at room temperature. Staining solution was prepared with stock X-gal solution according to the manufacturer’s protocol and added to each sample. Samples were incubated during 20 h at 37 °C without CO_2_. Cells were then rinsed with PBS and counterstained with Eosin (Sigma-Aldrich) during 8 min. Cells were viewed under a light confocal microscope and cells stained with blue were counted as senescent.

### High-throughput screening

High-throughput screening was conducted on CLARIOstar Plate Reade (BMG Labtech). VSMCs-HGPS were seeded in 38 μl of culture medium into black 384-well clear bottom plates. One day after seeding, 5 µM of compounds from chemical libraries were transferred into cell assay plates. In each plate, negative control (0.1% DMSO) and positive control (1 µM FK 866) were added in columns 1–23 and 2–24, respectively. Plates were then incubated for 2 days and processed for NAD^+^ quantification. To prevent the discovery of toxic molecules, the cell number was quantified in parallel by counting Hoechst-stained cells per field and candidates showing mortality superior to 55% were excluded.

### Statistics

Statistical analyses were performed with GraphPad Prism 8 software (GraphPad Software, Inc. San Diego, CA, USA). Screening Analysis was performed on Discngine Assay associated with TIBCO SpotFire software Statistical parameters including the definitions and exact value of n (e.g., total number of experiments, animals, cells), *p* values, and the types of the statistical tests are reported in the figure and corresponding figure legends. For the comparison of WT versus *Lmna*^*G609G/G609G*^ mice, we used unpaired *t* test, Wilcoxon–Mann–Whitney, and for the comparison on treated groups of animals we used Kruskall–Wallis or ANOVA test. Data are shown as mean ± standard error of mean unless other specification.

## Supplementary information

supplemental material

ORIGINAL DATSET

## Data Availability

All data supporting this study are presented in this published article and in its Supplementary information files.

## References

[1] De Sandre-Giovannoli A, Bernard R, Cau P, Navarro C, Amiel J, Boccaccio I, et al. Lamin A truncation in Hutchinson-Gilford progeria. Science. 2003;300:2055. 12702809 10.1126/science.1084125

[2] Eriksson M, Brown WT, Gordon LB, Glynn MW, Singer J, Scott L, et al. Recurrent de novo point mutations in lamin A cause Hutchinson-Gilford progeria syndrome. Nature. 2003;423:293–8. 12714972 10.1038/nature01629 PMC10540076

[3] Lin F, Worman HJ. Structural organization of the human gene encoding nuclear lamin A and nuclear lamin C. J Biol Chem. 1993;268:16321–6. 8344919

[4] Nigg EA, Kitten GT, Vorburger K. Targeting lamin proteins to the nuclear envelope: the role of CaaX box modifications. Biochem Soc Trans. 1992;20:500–4. 1397650 10.1042/bst0200500

[5] Ibrahim MX, Sayin VI, Akula MK, Liu M, Fong LG, Young SG, et al. Targeting isoprenylcysteine methylation ameliorates disease in a mouse model of progeria. Science. 2013;340:1330–3. 23686339 10.1126/science.1238880 PMC4295631

[6] Chen X, Yao H, Kashif M, Revêchon G, Eriksson M, Hu J, et al. A small-molecule ICMT inhibitor delays senescence of Hutchinson-Gilford progeria syndrome cells. eLife. 2021;10:e63284. 33526168 10.7554/eLife.63284 PMC7853716

[7] Carrero D, Soria-Valles C, López-Otín C. Hallmarks of progeroid syndromes: lessons from mice and reprogrammed cells. Dis Model Mech. 2016;9:719–35. 27482812 10.1242/dmm.024711 PMC4958309

[8] Gordon LB, Massaro J, D’Agostino RB, Campbell SE, Brazier J, Brown WT, et al. Impact of farnesylation inhibitors on survival in Hutchinson-Gilford progeria syndrome. Circulation. 2014;130:27–34. 24795390 10.1161/CIRCULATIONAHA.113.008285 PMC4082404

[9] Stehbens WE, Wakefield SJ, Gilbert-Barness E, Olson RE, Ackerman J. Histological and ultrastructural features of atherosclerosis in progeria. Cardiovasc Pathol J Soc Cardiovasc Pathol. 1999;8:29–39. 10.1016/s1054-8807(98)00023-4 10722246

[10] Stehbens WE, Delahunt B, Shozawa T, Gilbert-Barness E. Smooth muscle cell depletion and collagen types in progeric arteries. Cardiovasc Pathol J Soc Cardiovasc Pathol. 2001;10:133–6. 10.1016/s1054-8807(01)00069-2 11485857

[11] Olive M, Harten I, Mitchell R, Beers JK, Djabali K, Cao K, et al. Cardiovascular pathology in Hutchinson-Gilford progeria: correlation with the vascular pathology of aging. Arterioscler Thromb Vasc Biol. 2010;30:2301–9. 20798379 10.1161/ATVBAHA.110.209460 PMC2965471

[12] Osorio FG, Navarro CL, Cadiñanos J, López-Mejía IC, Quirós PM, Bartoli C, et al. Splicing-directed therapy in a new mouse model of human accelerated aging. Sci Transl Med. 2011;3:106ra107. 22030750 10.1126/scitranslmed.3002847

[13] Varga R, Eriksson M, Erdos MR, Olive M, Harten I, Kolodgie F, et al. Progressive vascular smooth muscle cell defects in a mouse model of Hutchinson-Gilford progeria syndrome. Proc Natl Acad Sci USA. 2006;103:3250–5. 16492728 10.1073/pnas.0600012103 PMC1413943

[14] Verdin E. NAD^+^ in aging, metabolism, and neurodegeneration. Science. 2015;350:1208–13. 26785480 10.1126/science.aac4854

[15] Chambon P, Weill JD, Mandel P. Nicotinamide mononucleotide activation of new DNA-dependent polyadenylic acid synthesizing nuclear enzyme. Biochem Biophys Res Commun. 1963;11:39–43. 14019961 10.1016/0006-291x(63)90024-x

[16] Revollo JR, Grimm AA, Imai S. The NAD biosynthesis pathway mediated by nicotinamide phosphoribosyltransferase regulates Sir2 activity in mammalian cells. J Biol Chem. 2004;279:50754–63. 15381699 10.1074/jbc.M408388200

[17] Bieganowski P, Brenner C. Discoveries of nicotinamide riboside as a nutrient and conserved NRK genes establish a Preiss-Handler independent route to NAD+ in fungi and humans. Cell. 2004;117:495–502. 15137942 10.1016/s0092-8674(04)00416-7

[18] Bai P. Biology of Poly(ADP-Ribose) polymerases: the factotums of cell maintenance. Mol Cell. 2015;58:947–58. 26091343 10.1016/j.molcel.2015.01.034

[19] Kim MY, Zhang T, Kraus WL. Poly(ADP-ribosyl)ation by PARP-1: “PAR-laying” NAD+ into a nuclear signal. Genes Dev. 2005;19:1951–67. 16140981 10.1101/gad.1331805

[20] Murata MM, Kong X, Moncada E, Chen Y, Imamura H, Wang P, et al. NAD+ consumption by PARP1 in response to DNA damage triggers metabolic shift critical for damaged cell survival. Mol Biol Cell. 2019;30:2584–97. 31390283 10.1091/mbc.E18-10-0650 PMC6740200

[21] Pitrez PR, Estronca L, Monteiro LM, Colell G, Vazão H, Santinha D, et al. Vulnerability of progeroid smooth muscle cells to biomechanical forces is mediated by MMP13. Nat Commun. 2020;11:4110. 32807790 10.1038/s41467-020-17901-2 PMC7431909

[22] Heidelberger C, Anderson SW. Fluorinated pyrimidines. xxi. the tumor-inhibitory activity of 5-trifluoromethyl-2’-deoxyuridine. Cancer Res. 1964;24:1979–85. 14247510

[23] Dexter DL, Wolberg WH, Ansfield FJ, Helson L, Heidelberger C. The clinical pharmacology of 5-trifluoromethyl-2’-deoxyuridine. Cancer Res. 1972;32:247–53. 4333494

[24] Hamczyk MR, Villa-Bellosta R, Gonzalo P, Andrés-Manzano MJ, Nogales P, Bentzon JF, et al. Vascular smooth muscle-specific progerin expression accelerates atherosclerosis and death in a mouse model of Hutchinson-Gilford Progeria syndrome. Circulation. 2018;138:266–82. 29490993 10.1161/CIRCULATIONAHA.117.030856 PMC6075893

[25] Villa-Bellosta R, Rivera-Torres J, Osorio FG, Acín-Pérez R, Enriquez JA, López-Otín C, et al. Defective extracellular pyrophosphate metabolism promotes vascular calcification in a mouse model of Hutchinson-Gilford progeria syndrome that is ameliorated on pyrophosphate treatment. Circulation. 2013;127:2442–51. 23690466 10.1161/CIRCULATIONAHA.112.000571

[26] Ansari HR, Raghava GPS. Identification of NAD interacting residues in proteins. BMC Bioinform. 2010;11:160. 10.1186/1471-2105-11-160 PMC2853471 20353553

[27] Anderson RM, Bitterman KJ, Wood JG, Medvedik O, Sinclair DA. Nicotinamide and PNC1 govern lifespan extension by calorie restriction in Saccharomyces cerevisiae. Nature. 2003;423:181–5. 12736687 10.1038/nature01578 PMC4802858

[28] Lin S-J, Kaeberlein M, Andalis AA, Sturtz LA, Defossez P-A, Culotta VC, et al. Calorie restriction extends Saccharomyces cerevisiae lifespan by increasing respiration. Nature. 2002;418:344–8. 12124627 10.1038/nature00829

[29] Massudi H, Grant R, Braidy N, Guest J, Farnsworth B, Guillemin GJ. Age-associated changes in oxidative stress and NAD+ metabolism in human tissue. PloS ONE. 2012;7:e42357. 22848760 10.1371/journal.pone.0042357 PMC3407129

[30] Zhang H, Ryu D, Wu Y, Gariani K, Wang X, Luan P, et al. NAD^+^ repletion improves mitochondrial and stem cell function and enhances life span in mice. Science. 2016;352:1436–43. 27127236 10.1126/science.aaf2693

[31] Covarrubias AJ, Perrone R, Grozio A, Verdin E. NAD+ metabolism and its roles in cellular processes during ageing. Nat Rev Mol Cell Biol. 2021;22:119–41. 33353981 10.1038/s41580-020-00313-x PMC7963035

[32] Rajman L, Chwalek K, Sinclair DA. Therapeutic potential of NAD-boosting molecules: the in vivo evidence. Cell Metab 2018;27:529–47. 29514064 10.1016/j.cmet.2018.02.011 PMC6342515

[33] Zhang H, Xiong Z-M, Cao K. Mechanisms controlling the smooth muscle cell death in progeria via down-regulation of poly(ADP-ribose) polymerase 1. Proc Natl Acad Sci USA. 2014;111:E2261–2270. 24843141 10.1073/pnas.1320843111 PMC4050581

[34] Watson A, Nong Z, Yin H, O’Neil C, Fox S, Balint B, et al. Nicotinamide Phosphoribosyltransferase in smooth muscle cells maintains genome integrity, resists aortic medial degeneration, and is suppressed in human thoracic aortic aneurysm disease. Circ Res. 2017;120:1889–902. 28356339 10.1161/CIRCRESAHA.116.310022

[35] Cobb AM, Larrieu D, Warren DT, Liu Y, Srivastava S, Smith AJO, et al. Prelamin A impairs 53BP1 nuclear entry by mislocalizing NUP153 and disrupting the Ran gradient. Aging Cell. 2016;15:1039–50. 27464478 10.1111/acel.12506 PMC5114580

[36] Liu Y, Drozdov I, Shroff R, Beltran LE, Shanahan CM. Prelamin A accelerates vascular calcification via activation of the DNA damage response and senescence-associated secretory phenotype in vascular smooth muscle cells. Circ Res. 2013;112:e99–109. 23564641 10.1161/CIRCRESAHA.111.300543

[37] Curtin NJ, Szabo C. Poly(ADP-ribose) polymerase inhibition: past, present and future. Nat Rev Drug Discov. 2020;19:711–36. 32884152 10.1038/s41573-020-0076-6

[38] Scheibye-Knudsen M, Mitchell SJ, Fang EF, Iyama T, Ward T, Wang J, et al. A high-fat diet and NAD(+) activate Sirt1 to rescue premature aging in cockayne syndrome. Cell Metab. 2014;20:840–55. 25440059 10.1016/j.cmet.2014.10.005 PMC4261735

[39] Guilbert SM, Cardoso D, Lévy N, Muchir A, Nissan X. Hutchinson-Gilford progeria syndrome: rejuvenating old drugs to fight accelerated ageing. Methods. 2021;190:3–12. 10.1016/j.ymeth.2020.04.005 32278808

[40] Koblan LW, Erdos MR, Wilson C, Cabral WA, Levy JM, Xiong Z-M, et al. In vivo base editing rescues Hutchinson-Gilford progeria syndrome in mice. Nature. 2021;589:608–14. 33408413 10.1038/s41586-020-03086-7 PMC7872200

[41] Santiago-Fernández O, Osorio FG, Quesada V, Rodríguez F, Basso S, Maeso D, et al. Development of a CRISPR/Cas9-based therapy for Hutchinson-Gilford progeria syndrome. Nat Med. 2019;25:423–6. 30778239 10.1038/s41591-018-0338-6 PMC6546610

[42] Beyret E, Liao H-K, Yamamoto M, Hernandez-Benitez R, Fu Y, Erikson G, et al. Single-dose CRISPR-Cas9 therapy extends lifespan of mice with Hutchinson-Gilford progeria syndrome. Nat Med 2019;25:419–22. 30778240 10.1038/s41591-019-0343-4 PMC6541418

[43] Dhillon S. Lonafarnib: first approval. Drugs. 2021;81:283–9. 33590450 10.1007/s40265-020-01464-z PMC7985116

[44] Gordon LB, Shappell H, Massaro J, D’Agostino RB, Brazier J, Campbell SE, et al. Association of lonafarnib treatment vs no treatment with mortality rate in patients with Hutchinson-Gilford progeria syndrome. JAMA. 2018;319:1687–95. 29710166 10.1001/jama.2018.3264 PMC5933395

[45] Trammell SAJ, Schmidt MS, Weidemann BJ, Redpath P, Jaksch F, Dellinger RW, et al. Nicotinamide riboside is uniquely and orally bioavailable in mice and humans. Nat Commun. 2016;7:12948. 27721479 10.1038/ncomms12948 PMC5062546

[46] Heilbronn LK. Clinical trials corner. Nutr Healthy Aging. 2017;4:193–4. 28447073 10.3233/NHA-170001 PMC5389020

[47] Owens, GK, Loeb, A, Gordon, D, Thompson, MM. Expression of smooth muscle-specific a-isoactin in cultured vascular smooth muscle cells: relationship between growth and cytodifferentiation. J Cell Biol.1986;102:343–52. 10.1083/jcb.102.2.343 PMC2114077 3944187

[48] Lo Cicero A, Jaskowiak A-L, Egesipe A-L, Tournois J, Brinon B, Pitrez PR, et al. A high throughput phenotypic Screening reveals compounds that counteract premature osteogenic differentiation of HGPS iPS-derived mesenchymal stem cells. Sci Rep. 2016;6:34798. 27739443 10.1038/srep34798 PMC5064407

[49] Bernofsky C, Swan M. An improved cycling assay for nicotinamide adenine dinucleotide. Anal Biochem. 1973;53:452–8. 4351948 10.1016/0003-2697(73)90094-8

