# Supplementary material for: Inhibition of poly(ADP-Ribosyl)ation reduced vascular smooth muscle cells loss and improves aortic disease in a mouse model of human accelerated aging syndrome
Source: Cell Death Dis. 2024 Oct 2;15(10):723. doi: 10.1038/s41419-024-07078-7 (PMC11448498; doi:10.1038/s41419-024-07078-7)
Supplement: Supplementary file 1 — supplemental material [file 41419_2024_7078_MOESM1_ESM.docx]

**SUPPLEMENTAL INFORMATION**

**Table S1: Compounds selected after dose-response screening**

|  | **Screening** | | | | **Second test** | | **DRs results** | | |
| --- | --- | --- | --- | --- | --- | --- | --- | --- | --- |
|  | **% activity** | **% viability** | **PZS**  **% activity** | **RZS**  **% activity** | **% activity** | **% viability** | **DRs validated** | **EC50 μM** | **% viability at EC50** |
| ***Trifluridine*** | 419.22 | 35.05 | 7.64 | 7.94 | 183.24 | 50.2 | yes | 0.44 | 70 |
| ***Methotrexate*** | 329.06 | 62.13 | 4.21 | 5.71 | 192.09 | 50.29 | yes | 3 | 50 |
| ***Pemetrexed disodium*** | 290.97 | 62.54 | 4.68 | 4.77 | 207.13 | 49.48 | yes | 0.6 | 60 |
| ***Tenovin-1*** | 301.71 | 58.68 | 5.13 | 5.04 | 109.76 | 95.77 | no | - | - |
| ***Proguanil hydrochloride*** | 393.35 | 41.88 | 7.05 | 7.3 | 110.97 | 96.94 | no | - | - |
| ***Ibandronate*** | 413.28 | 30.78 | 7.82 | 7.79 | 90.49 | 103.54 | no | - | - |
| ***Diflunisal*** | 509.55 | 40.31 | 7.75 | 10.17 | 101.84 | 103.21 | no | - | - |
| ***Dextromethorphan hydrobromide monohydrate*** | 343.41 | 35.61 | 7.76 | 6.07 | 98.13 | 110.2 | no | - | - |

**Table S2: Listing of primers used in this study**

| **PRIMARY ANTIBODIES** | **HOST** | **REFERENCE** | **DILUTION** |
| --- | --- | --- | --- |
| α-SMA | mouse | Sigma #AS228 | 1/500 |
| AIF | rabbit | Abcam #ab32516 | 1/1,000 |
| CytC | mouse | Abcam #ab13575 | 1/200 |
| GAPDH | mouse | Sigma #MAB374 | 1/10,000 |
| γH2AX | rabbit | Cell Signaling #2577 | 1/500 |
| Lamin A/C | mouse | Santa Cruz Biotechnology #sc-376248 | 1/500 |
| Nampt | rabbit | Abcam #ab5480 | 1/1,000 |
| NRK2 | rabbit | US Biological #037146 | 1/1,000 |
| PAR | mouse | Millipore Calbiochem #AM80 | 1/500 |
| PARP-1 | rabbit | Santa Cruz Biotechnology #sc-8007 | 1/1,000 |
| MFN-1 | rabbit | Abcam #ab14734 | 1/1,000 |
| p16 | rabbit | Abcam#ab211542 | 1/50 |
| NOX2 | rabbit | Abcam #ab310337 | 1/50 |
|  |  |  |  |
| **SECONDARY ANTIBODIES** | **HOST** | **REFERENCE** | **DILUTION** |
| StarBright IgG | goat | BioRad #12005867 | 1/10,000 |
| StarBright IgG | goat | BioRad #12004161 | 1/10,000 |
| Alexa Fluor 488 | goat | Invitrogen #A-11001 | 1/5,000 |
| Alexa Fluor 546 | goat | Invitrogen #A-11010 | 1/5,000 |


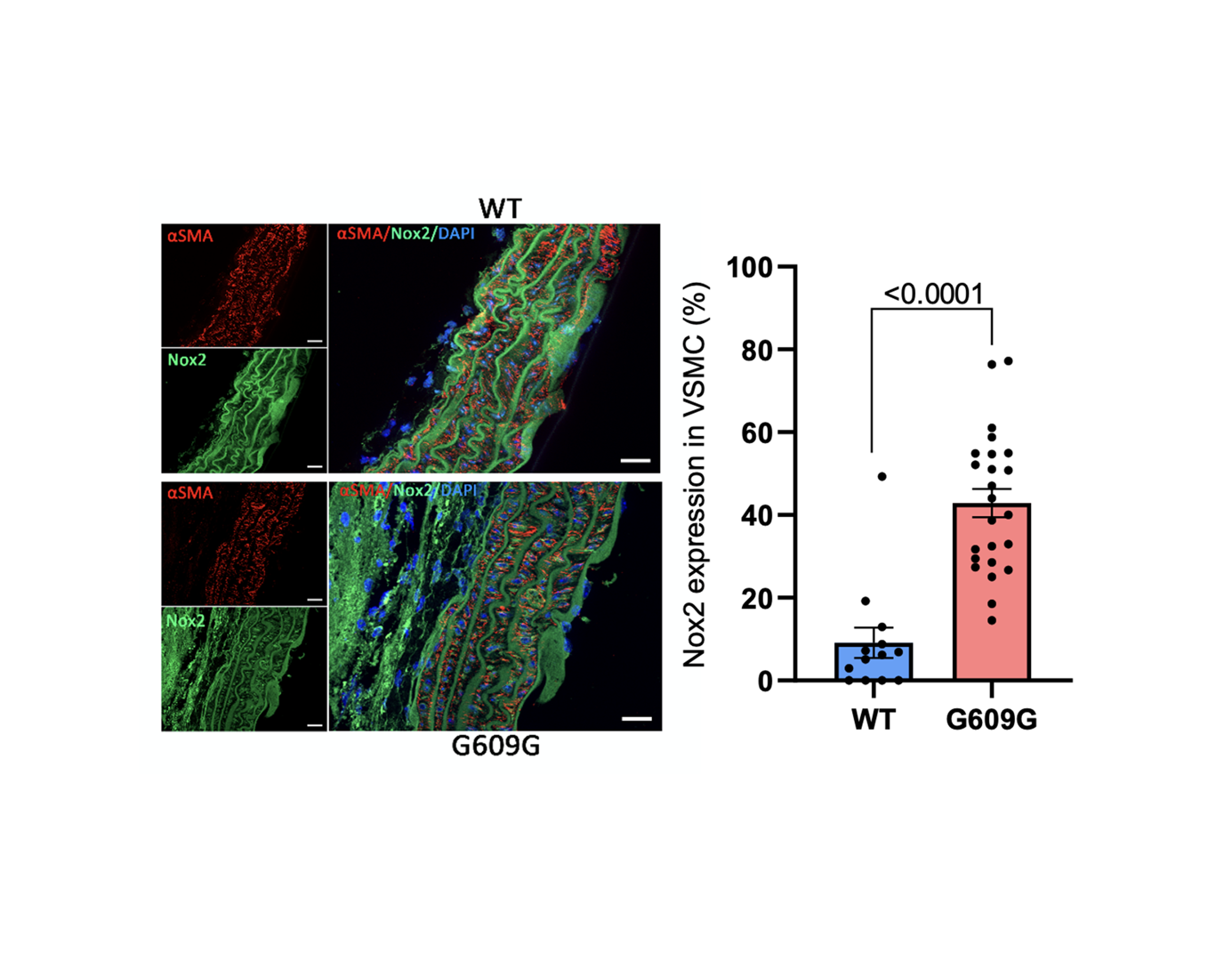


**Figure S1: Increased aortic oxidative stress in *Lmna^G609G/G609G^* mice.** Immunostaining of aorta cross sections from *Lmna^G609G/G609G^* (G606G) and WT mice showing α-SMA (Red) and Nox2 (Green) indicating oxidative stress in cells. Nuclei stained with DAPI (Blue). Scale bar, 20µm. Graph show the percentage of Nox2 positive VSMCs in aortic arch quantification in *Lmna^G609G/G609G^* (n = 6) and WT (n = 3) mice. Bars indicate mean ± standard error of mean and number above columns indicate p-values. Differences were analyzed by unpaired t-test.


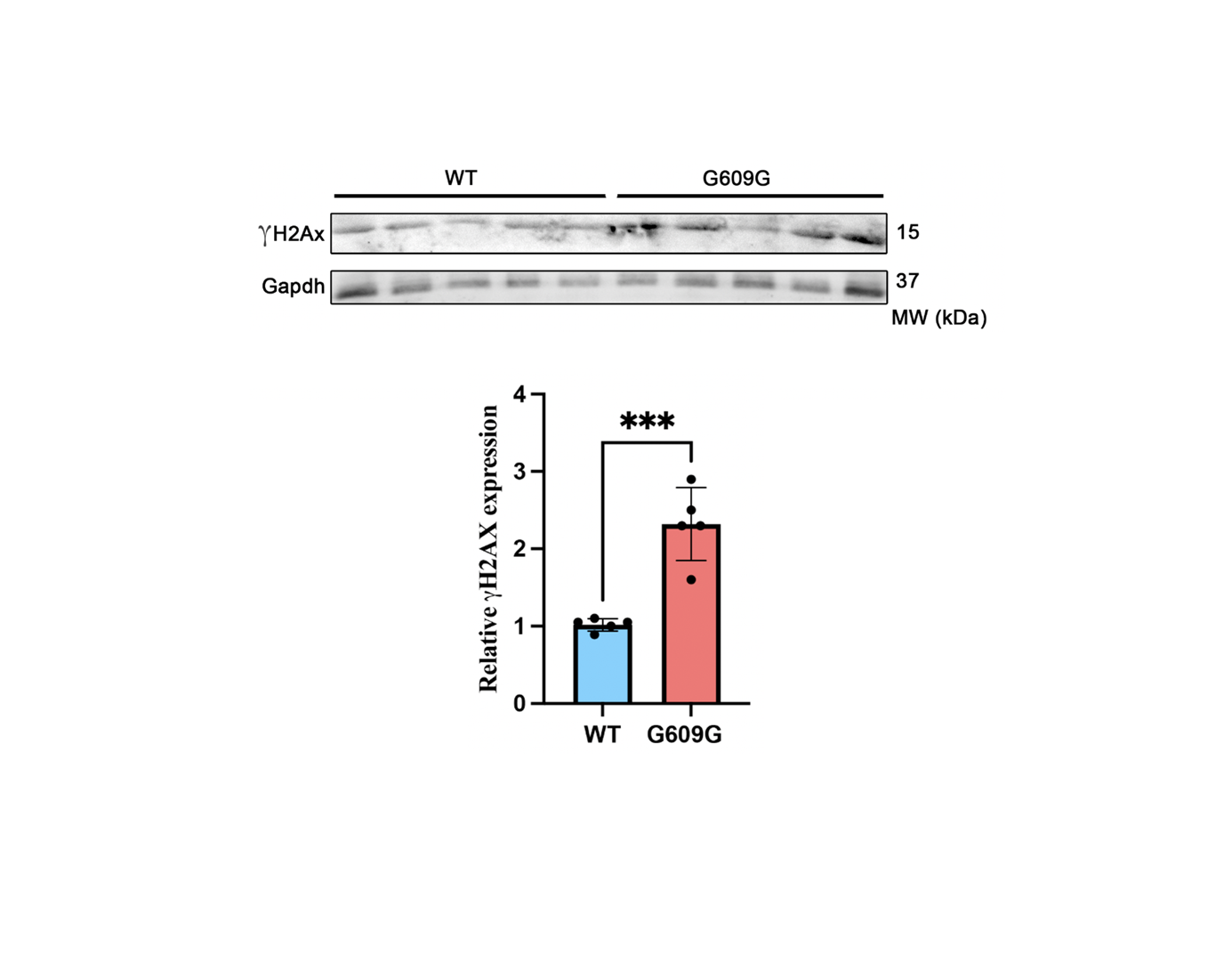


**Figure S2: Inefficient DNA repair mechanism in aorta from *Lmna^G609G/G609G^* mice.** (**A**) Representative immunoblots showing γH2Ax protein level in aorta from *Lmna^G609G/G609G^* (G609G) and WT mice. Gapdh was used for normalization. Graph shows γH2Ax expression level in *Lmna^G609G/G609G^* (n = 5) and WT (n = 5) mice. Bars indicate mean ± standard error of mean and numbers above columns indicate p-values. Differences were analyzed by unpaired t-test.

**Figure S3: Effect of Trifluridine on cell proliferation.** Bar graphs are representative of Ki67 staining of HGPS-VSMCs indicated as percentage in DMSO- (control -), Olaparib-(control +) and Trifluridine treated cells (n=3 experiments).

**Figure S4: Effect of Trifluridine and TAS-102 on HGPS-VSMCs.** Bar graphs are representative of NAD^+^ (NAD^+^ and NADH) and ATP content normalized by the cell number (AU) in DMSO- (control -), FK866-,Olaparib-(control +) or Trifluridine or TAS-102 treated HGPS-VSMCs.

**
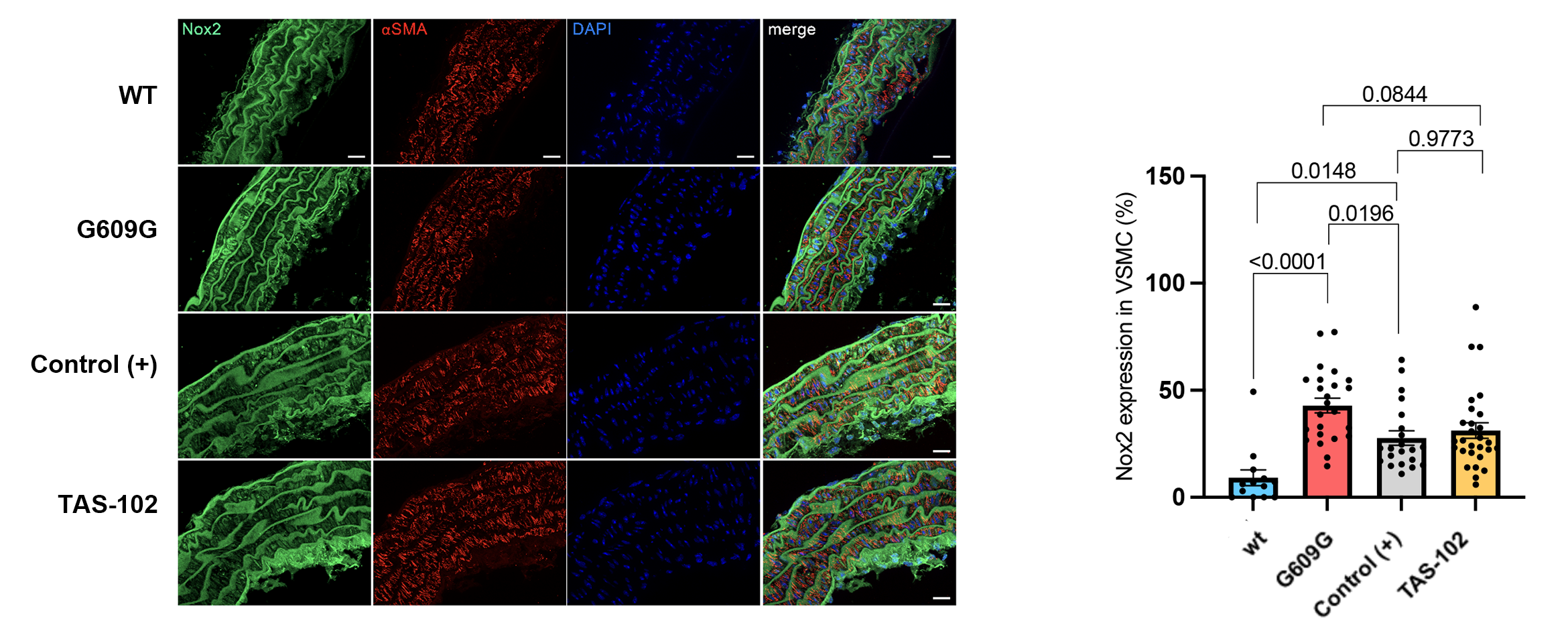
**

**Figure S5:** **TAS-102 treatment decreased oxidative stress in the media of the aorta from *Lmna^G609G/G609G^* mice.** Immunostaining of aorta cross sections from *Lmna^G609G/G609G^* (G609G) and WT mice showing α-SMA (Red) and Nox2 (Green) indicating oxidative stress in cells. Nuclei stained with DAPI (Blue). Scale bar, 20µm. Graph compare the percentage of Nox2 positive VSMCs in aortic arch quantification in *Lmna^G609G/G609G^* (n = 6) and treated *Lmna^G609G/G609G^*  with TAS-102 (n=6), Olaparib (n=5) versus WT (n = 3) mice. Bars indicate mean ± standard error of mean and number above columns indicate p-values. Differences were analyzed by one-way ANOVA test.

**
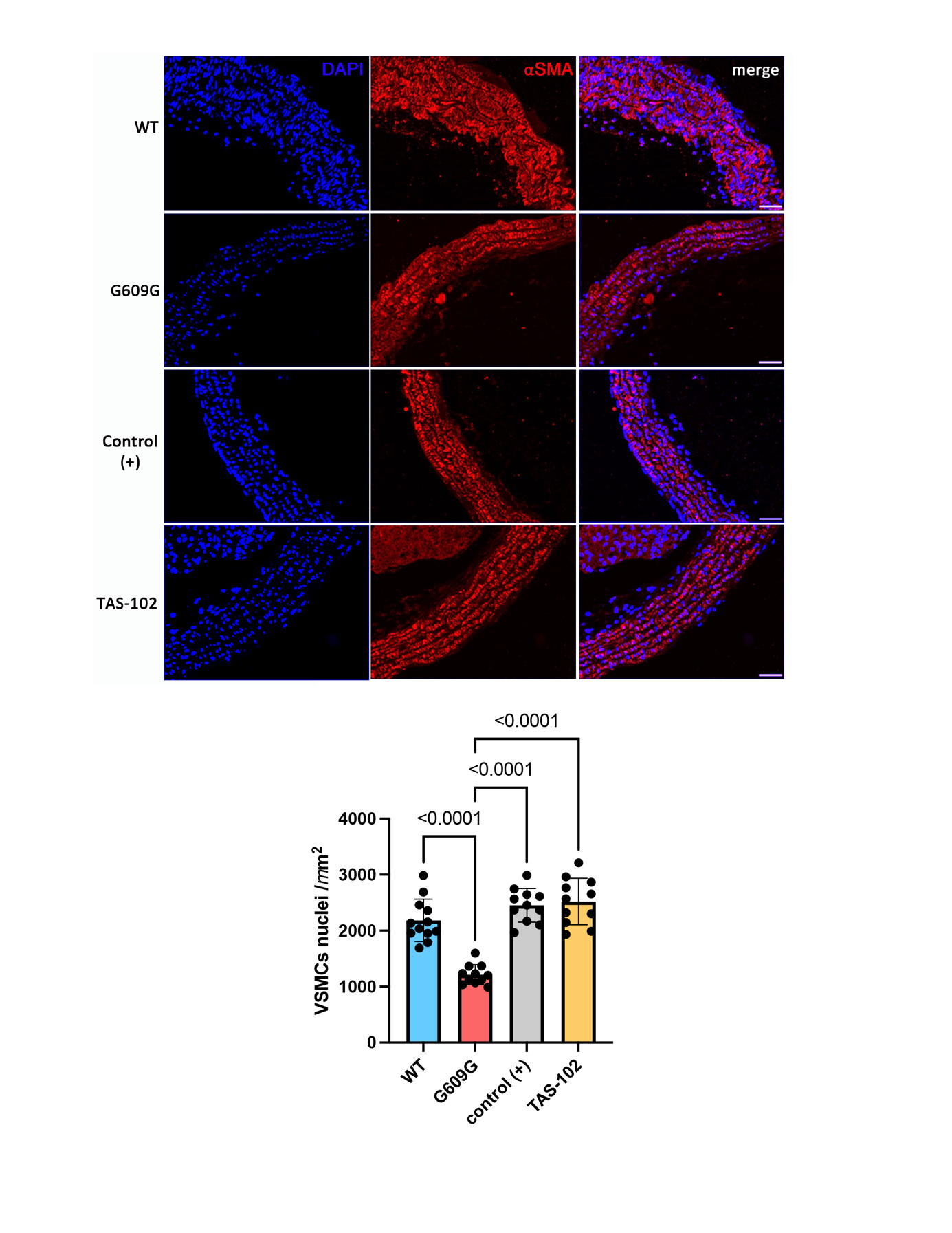
**

**Figure S6: TAS-102 treatment restores α-SMA positive cells in the media of the aorta of levels and improves aortic disease.** Representative aorta cross sections from *Lmna^G609G/G609G^*, WT and *Lmna^G609G/G609G^* mice treated with either TAS-102, Olaparib “Control (+)” or DMSO. Scale bar, 50µm. Bars indicate mean ± standard error of mean and numbers above columns indicate p-values. Difference was analyzed by one-way ANOVA followed by Tukey’s multiple comparison test.
