# Supplementary figures and images for: Inhibition of poly(ADP-Ribosyl)ation reduced vascular smooth muscle cells loss and improves aortic disease in a mouse model of human accelerated aging syndrome
Source: Cell Death Dis. 2024 Oct 2;15(10):723. doi: 10.1038/s41419-024-07078-7 (PMC11448498; doi:10.1038/s41419-024-07078-7)

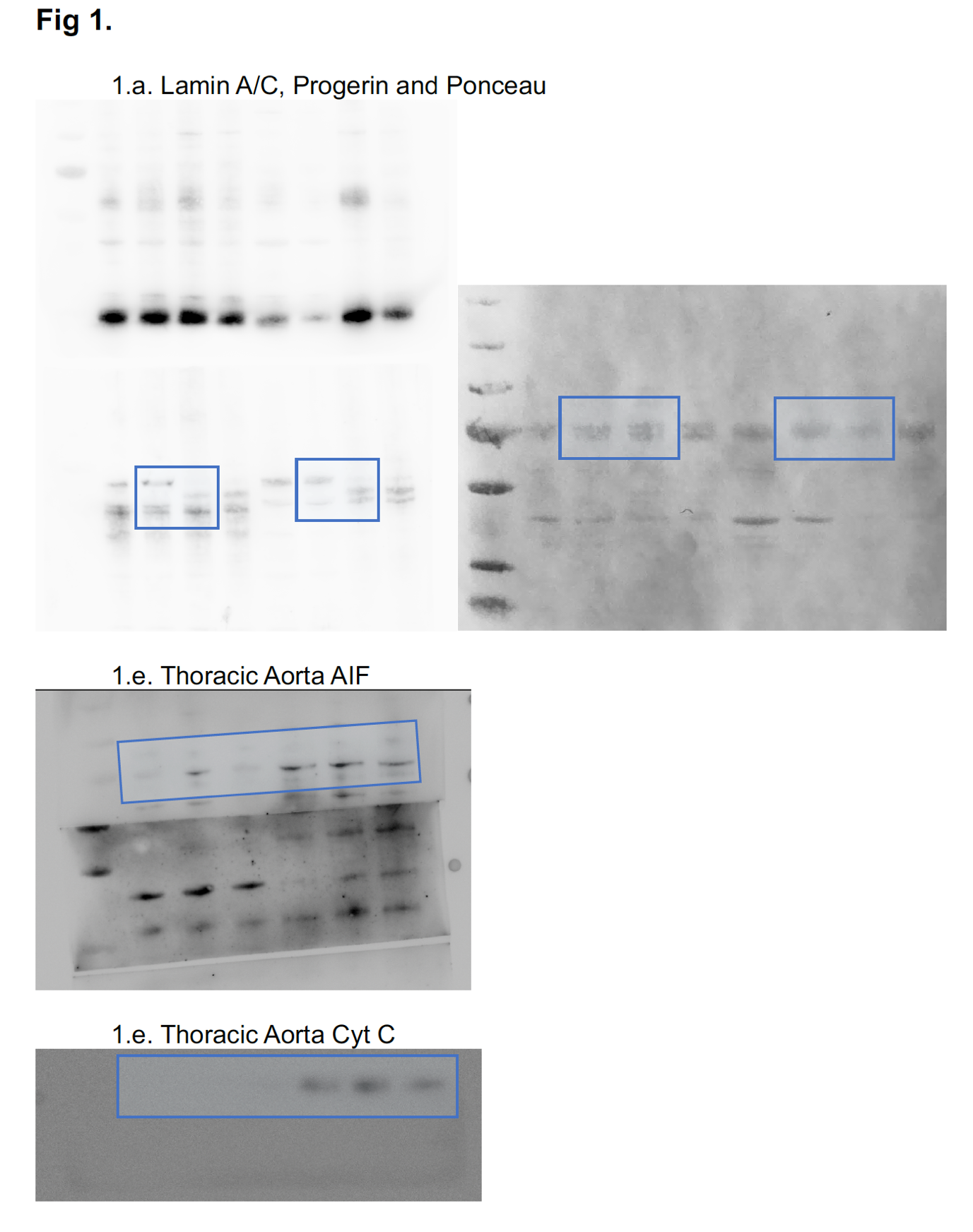


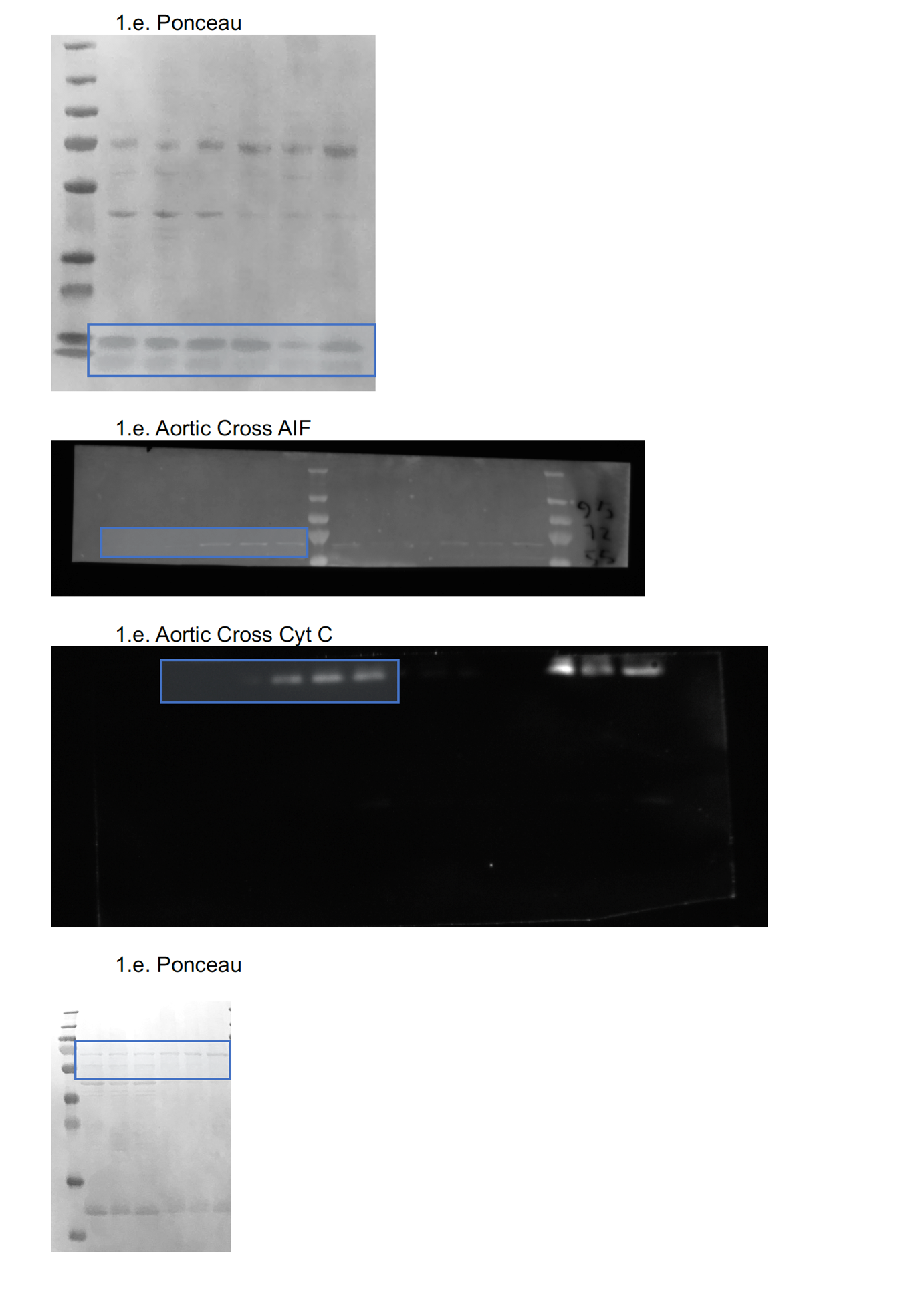


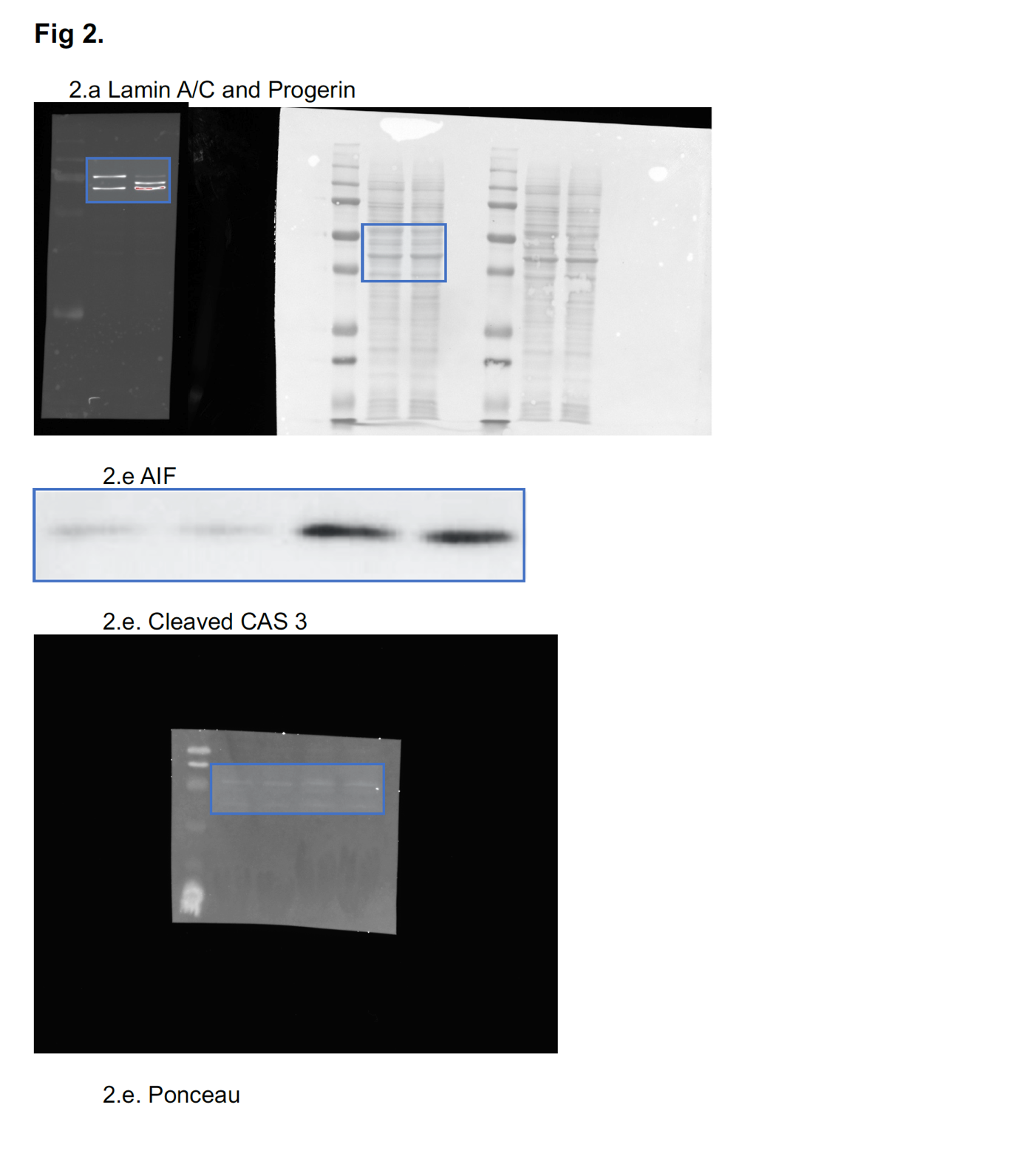


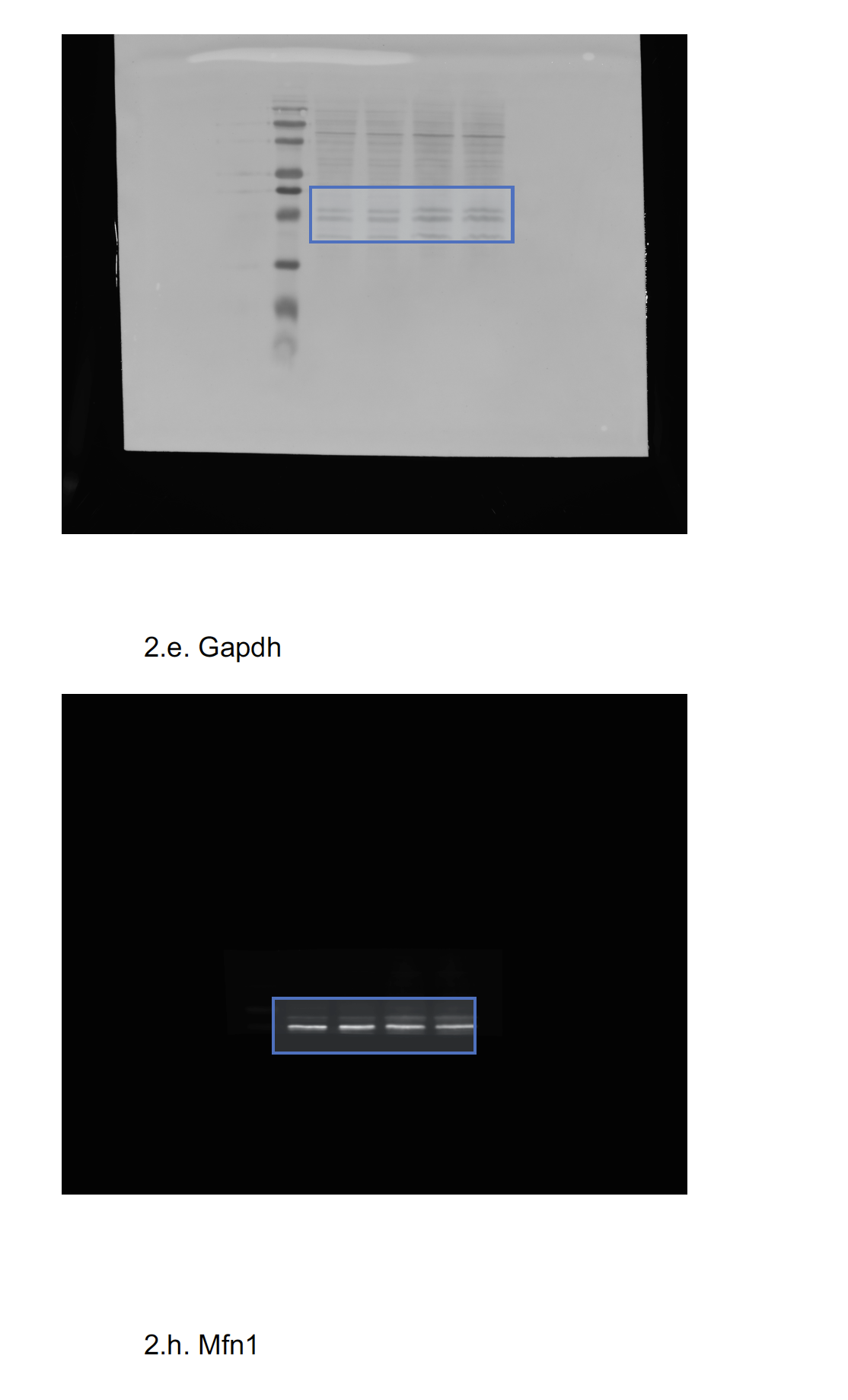


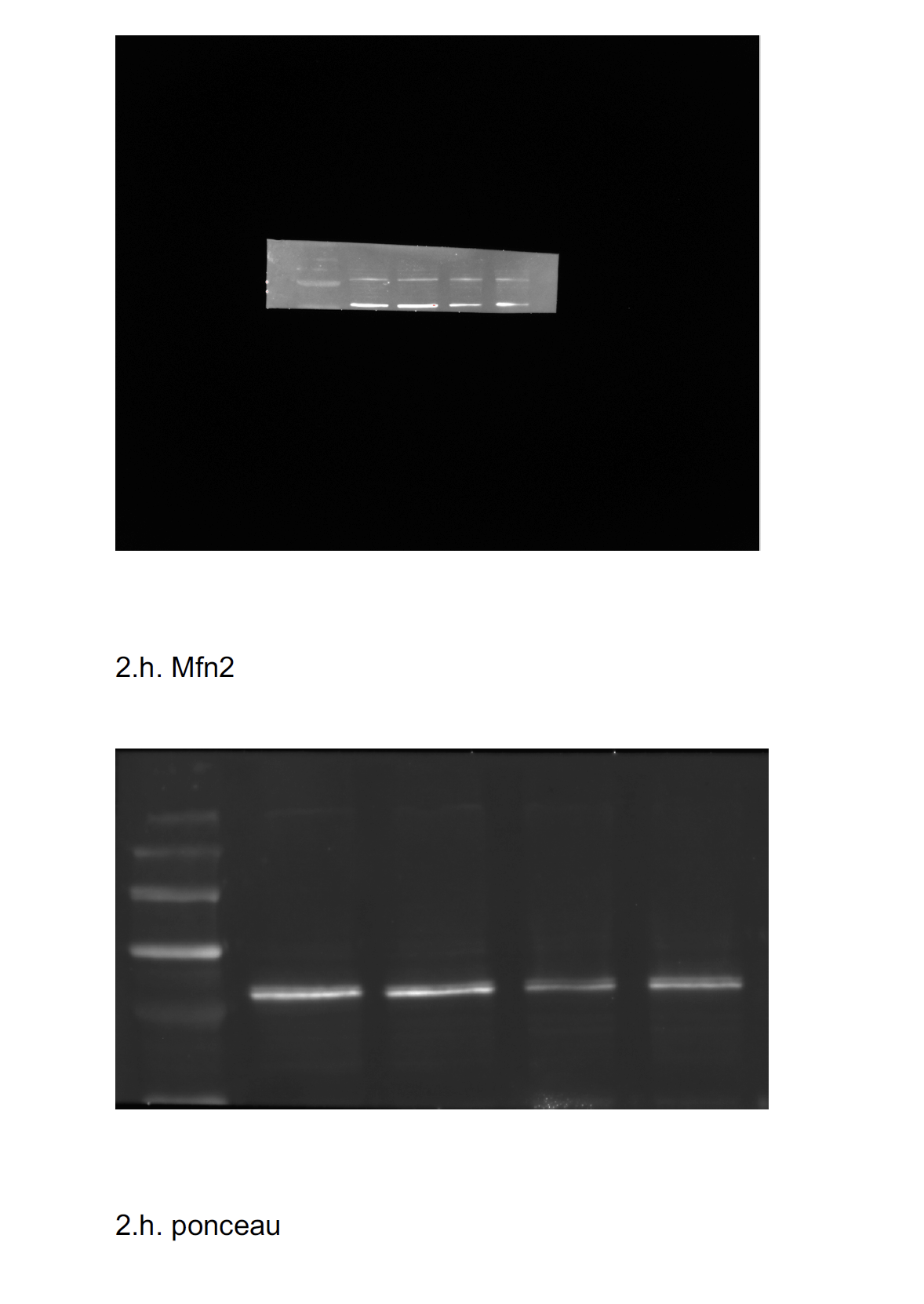


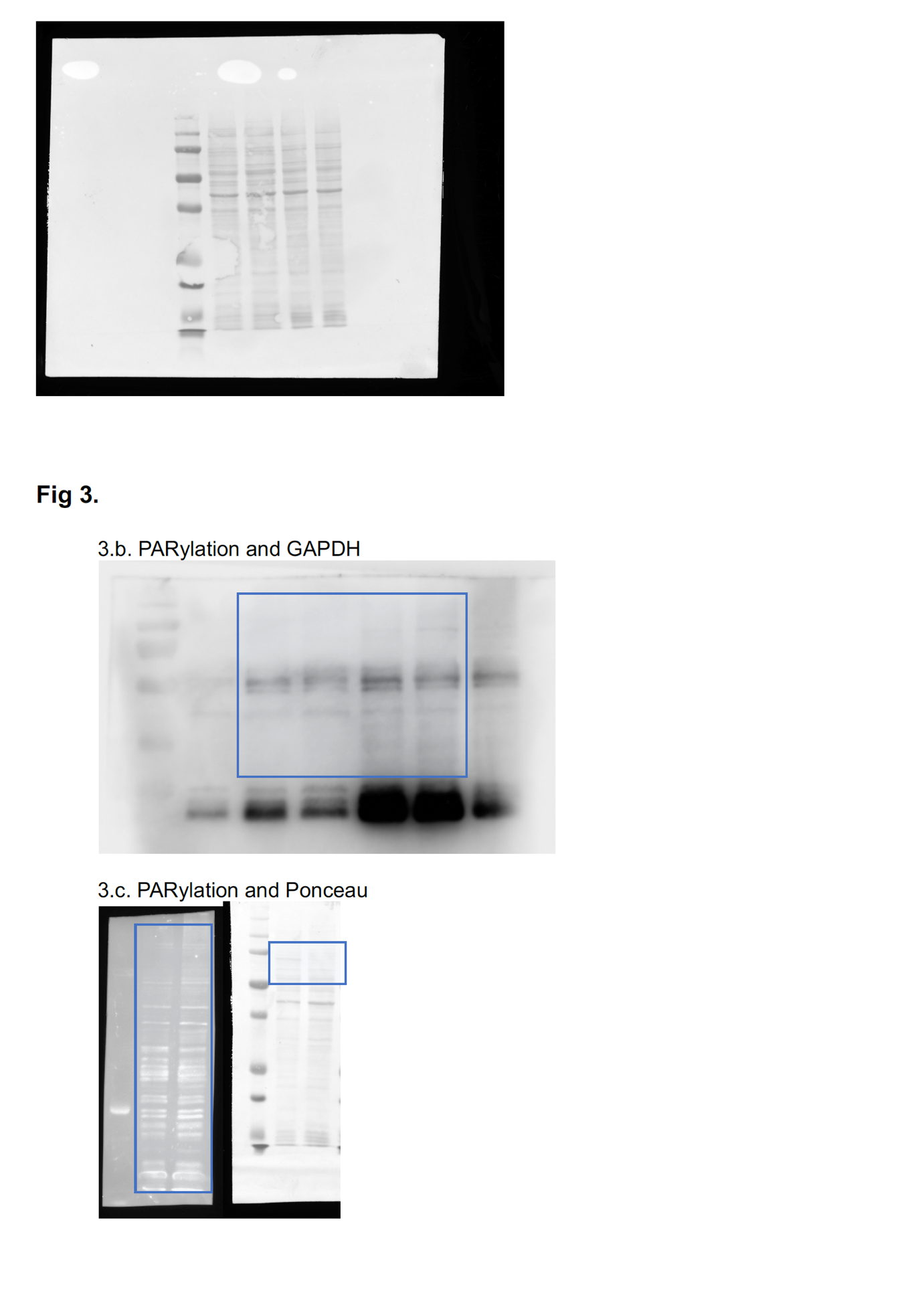


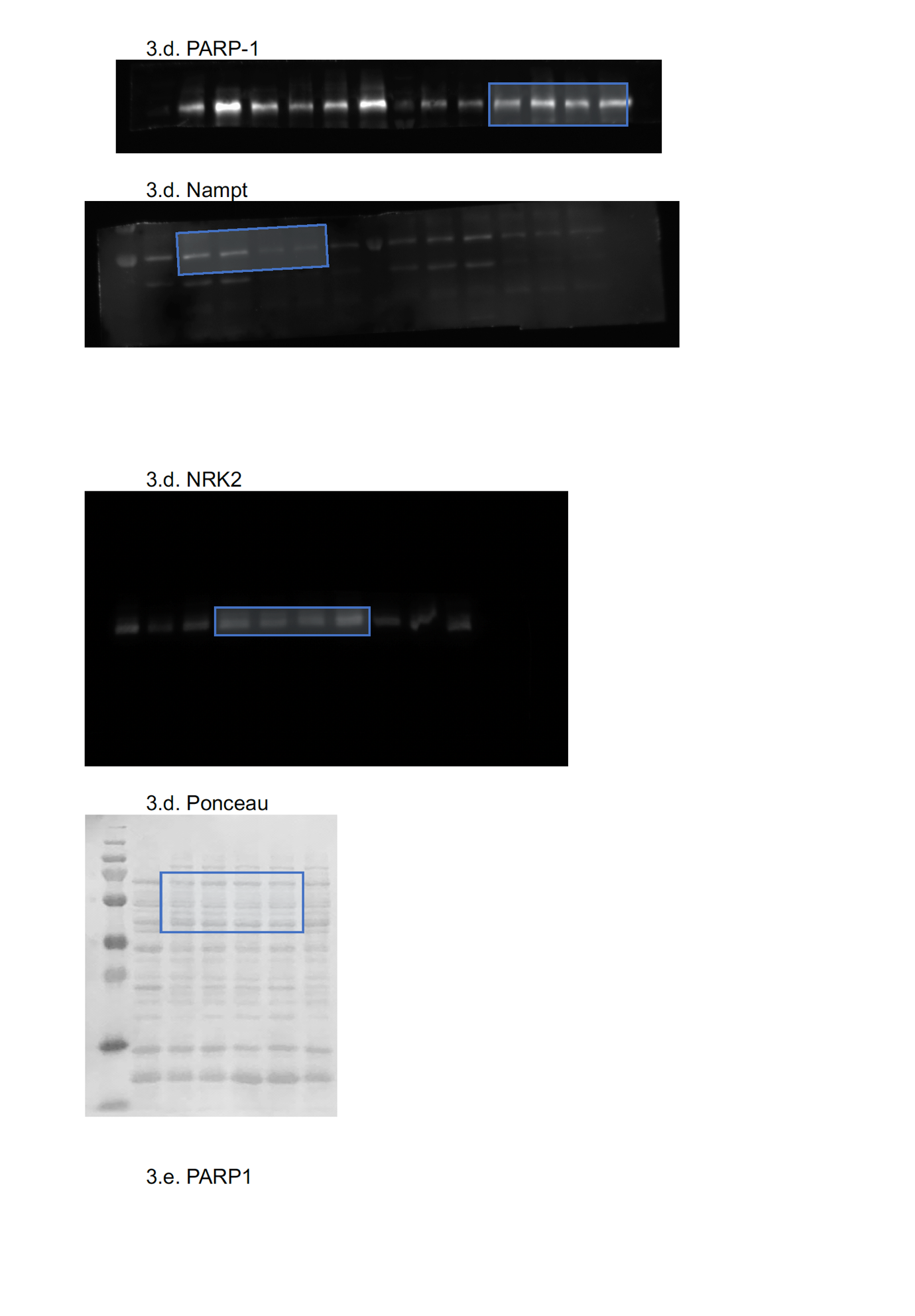


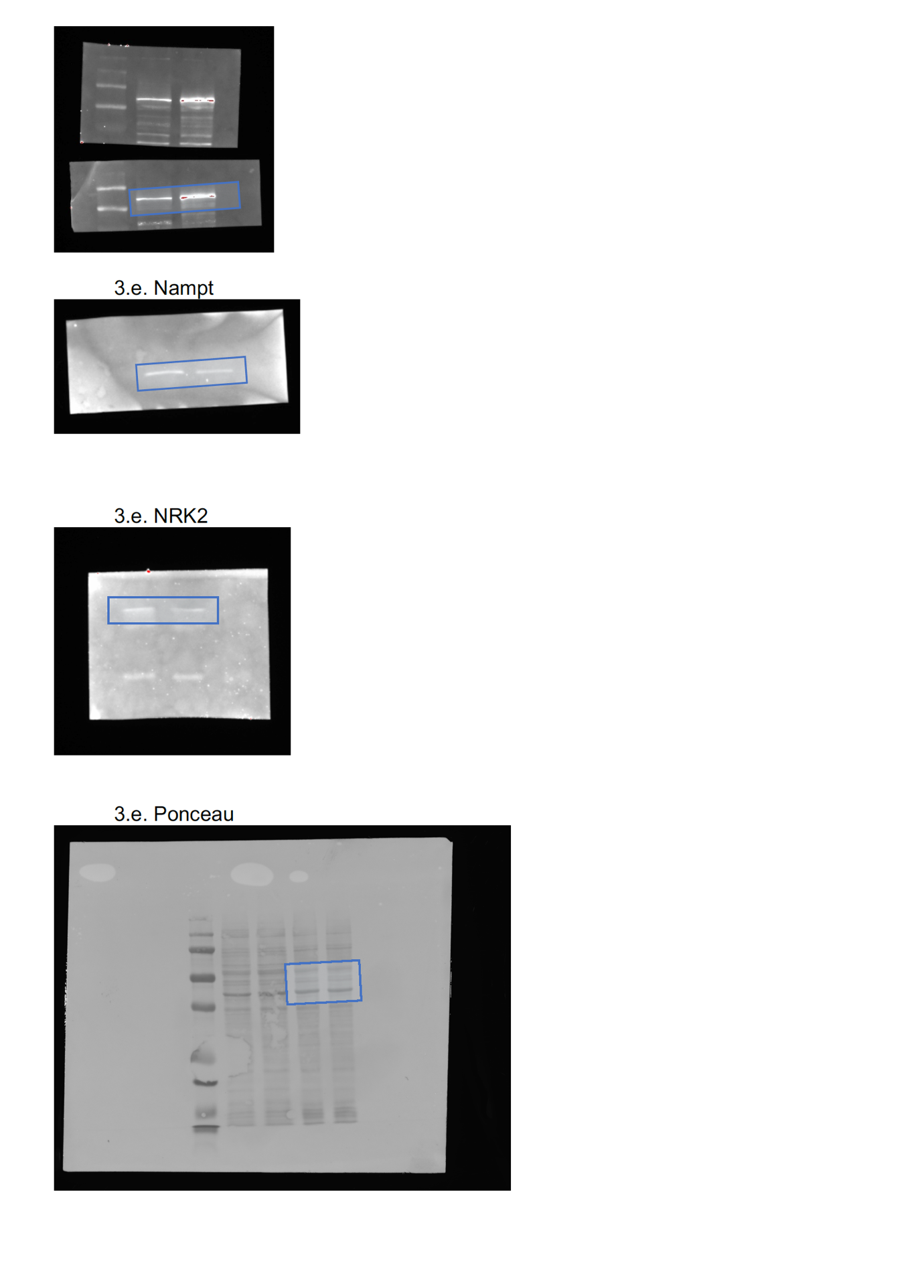


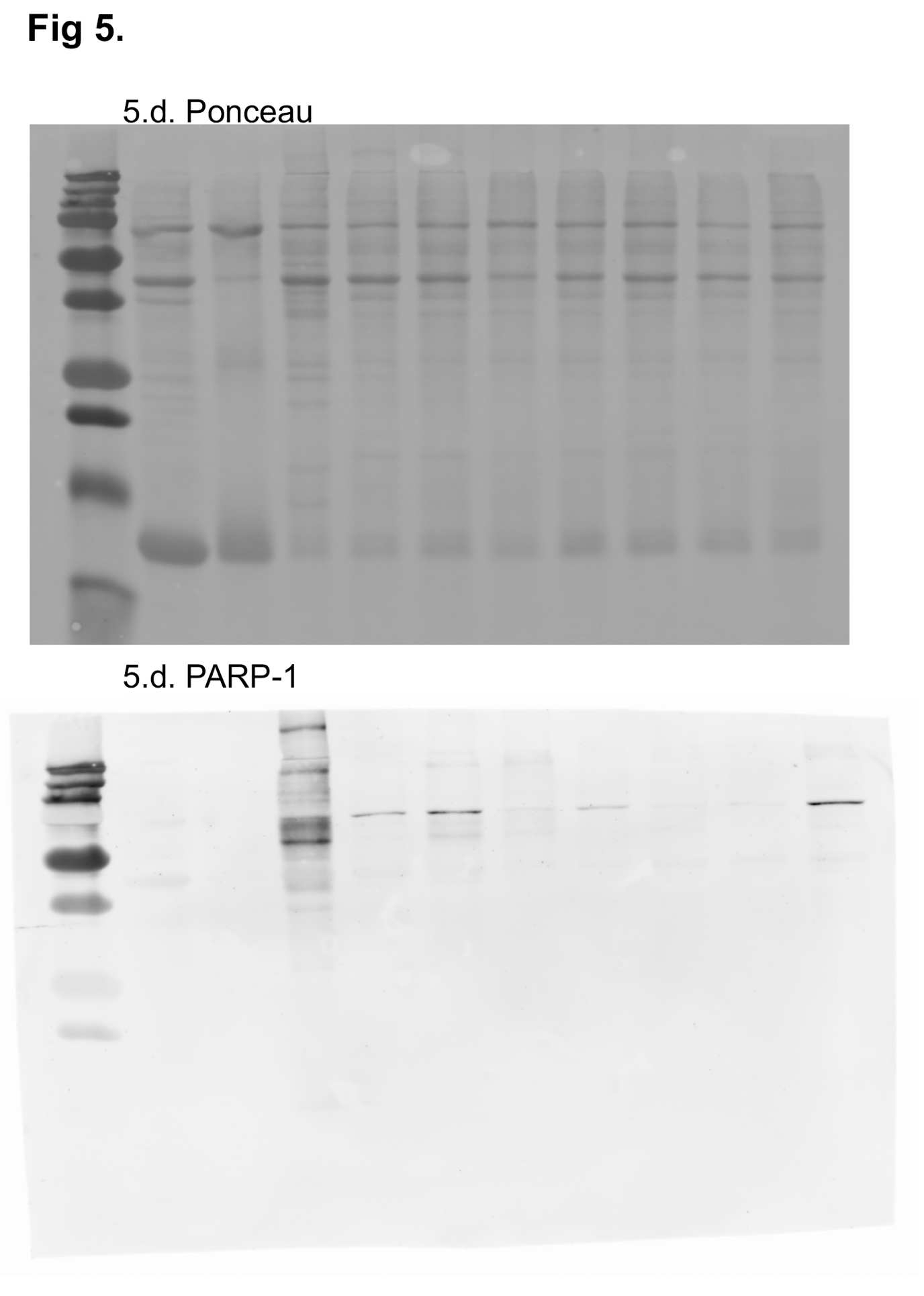

Supplement: Supplementary file 2 — ORIGINAL DATSET [file 41419_2024_7078_MOESM2_ESM.docx]
